# Fiber burden and asbestos-related diseases: an umbrella review

**DOI:** 10.1016/j.gaceta.2021.04.001

**Published:** 2021-06-11

**Authors:** José María Ramada Rodilla, Beatriz Calvo Cerrada, Consol Serra Pujadas, George L. Delclos, Fernando G. Benavides

**Affiliations:** a Institut Hospital del Mar d’Investigacions Mèdiques (IMIM), Barcelona, Spain; b Servei de Salut Laboral, Parc de Salut Mar, Barcelona, Spain; c Centro de Investigación Biomédica en Red de Epidemiología y Salud Pública (CIBERESP), Spain; d Center for Research in Occupational Health (CISAL-UPF), Barcelona, Spain; e Southwest Center for Occupational and Environmental Health, The University of Texas Health Science at Houston School of Public Health, Houston, Texas, USA

**Keywords:** Asbestos, Inorganic fibers, Exposure assessment, Mesothelioma, Pulmonary fibrosis, Lung cancer, Laryngeal cancer, Ovarian cancer, Gastrointestinal cancer, Asbesto, Fibras inorgánicas, Asesoramiento de exposición, Mesotelioma, Fibrosis pulmonar, Cáncer de pulmón, Cáncer de laringe, Cáncer de ovario, Cáncer gastrointestinal

## Abstract

**Objective:**

What are the levels of asbestos exposure that cause each type of health effect? The objective of this study was to review the available scientific evidence on exposure levels for asbestos and their relationship to health effects.

**Method:**

An umbrella review of English-language reviews and meta-analyses, from 1980 to March 2021 was conducted. We included reviews involving quantified asbestos exposures and health outcomes. The review has been adapted to the indications of the PRISMA declaration. Methodological quality of the selected studies was assessed using the AMSTAR instrument.

**Results:**

We retrieved 196 references. After applying the search strategy and quality analysis, 10 reviews were selected for in-depth analysis. For lung cancer, the highest risk was observed with exposure to amphiboles. Longer, thinner fibers had the greatest capacity to cause lung cancer, especially those > 10 μm in length. For mesothelioma, longer and thinner fibers were also more pathogenic; amphiboles ≥ 5 μm are especially associated with increased mesothelioma risk. No studies observed an increased risk for lung cancer or mesothelioma at asbestos exposure levels < 0.1 f/ml. No reviews provided information on exposure concentrations for pulmonary fibrosis. Currently, there is limited evidence in humans to establish the causal relationship between gastrointestinal cancer and asbestos exposure.

**Conclusions:**

Banning all asbestos exposure remains the best measure to preventing its negative health effects. The highest quality reviews and meta-analyses support that there is little risk of lung cancer or mesothelioma at daily exposure levels below 0.1 f/ml.

## RESUMEN

**Objetivo:**

Revisar la evidencia científica disponible sobre los niveles de exposición al asbesto y su relación con los efectos sobre la salud.

**Método:**

Se realizó una revisión de revisiones sistemáticas y metaanálisis en inglés, desde 1980 hasta marzo de 2021. Se incluyeron revisiones que involucran exposiciones cuantificadas al asbesto y resultados de salud. La revisión se adaptó a las indicaciones de la Declaración PRISMA. La calidad metodológica de los estudios seleccionados fue evaluada mediante el instrumento AMSTAR.

**Resultados:**

Se recuperaron 196 referencias y tras aplicar la estrategia de búsqueda y analizar la calidad se seleccionaron 10 revisiones para un análisis en profundidad. Para el cáncer de pulmón, se observó mayor riesgo con la exposición a anfíboles. Las fibras más largas y delgadas presentaron mayor capacidad de causar cáncer de pulmón, especialmente aquellas de longitud > 10 μm. Para el mesotelioma, las fibras más largas y delgadas también fueron más patógenas; los anfíboles ≥ 5 μm se asociaron con un mayor riesgo de mesotelioma. Ningún estudio observó mayor riesgo de cáncer de pulmón o de mesotelioma con niveles de exposición al asbesto < 0,1 f/ml. Ningún estudio proporcionó información sobre concentraciones de exposición para la fibrosis pulmonar. Actualmente existe evidencia limitada en humanos para establecer la relación causal entre la exposición al asbesto y el cáncer gastrointestinal.

**Conclusiones:**

Prohibir toda exposición al asbesto es la mejor medida para prevenir sus efectos negativos para la salud. Las revisiones y metaanálisis de más alta calidad respaldan que hay escaso riesgo de cáncer de pulmón y de mesotelioma con niveles de exposición diaria por debajo de 0,1 f/ml.

## Background

Asbestos is the generic name for a group of fibrous silicates present in nature, used in industrial processes and in the production of several products for domestic and environmental use.^1–4^ According to the chemical composition and physical properties, asbestos fibers are classified as serpentine (chrysotile or white asbestos), and amphibole comprising crocidolite (blue asbestos), amosite (brown asbestos), anthophyllite (yellow asbestos), tremolite (gray asbestos), and actinolite.^2,3,5^

It is well known that exposure to asbestos fibers can lead to diseases such as asbestosis (or diffuse interstitial pulmonary fibrosis), diffuse pleural fibrosis, rounded atelectasis (Blesovski’s syndrome), malignant mesothelioma and also ovary, lung and laryngeal cancer. Currently, there is limited evidence in humans to establish the causal relationship between gastrointestinal cancer and asbestos exposure.^1,3,6–8^

The health hazard of asbestos depends on several factors such as its concentration in the air, the exposure time, the type and the size of the fibers, the respiratory rate associated with physical exertion and thermo-hygrometric conditions, the anatomical and functional conditions of exposed workers and there exists a tobacco smoke modulating effect.^6^

The unit measures used for asbestos exposure may vary. The ACGIH establishes threshold limit values in occupational health.^9^ On the one hand, fibers per cubic centimeter (f/cc or f/cm^3^), equivalent to fibers per milliliter (f/ml), is commonly used to measure exposure during a working day (about 8 hours of duration) or during short-term exposures. These measures are compared with the permissible exposure limits (PEL, OSHA), the threshold limit values (TLV, ACGIH), the recommended exposure limits (REL, NIOSH) or the indicative occupational exposure limit values (IOELV, EU). On the other hand, fibers per milliliter and year (f/ml-y) is an exposure measure that represents the cumulative exposure to asbestos over a working life and is the measure that best reflects exposure intensity (exposure intensity equals the average concentration of asbestos in air multiplied by the duration of the exposure). Expressions such as fibers/milliliter-year (f/ml-y), fibers-year/milliliter (f-y/ml), fibers/cubic centimeter-year (f/cc-y) and fibers-year/cubic centimeter (f-y/cc) can be found in the literature. In order to analyze the effects of cancer, pulmonary fibrosis, mesothelioma and other pathologies, epidemiological studies tend to use this measure of cumulative exposure. Also, exposure units in millions of particles per cubic foot per year (mppcf-y) have been used.

The OSHA has set a PEL for asbestos at 0.1 fiber per cubic centimeter of air as an eight-hour time-weighted average (TWA), with an excursion limit (EL) of 1.0 asbestos fibers per cubic centimeter over a 30-minute period.^10^ Likewise, in Europe the 2009/148/EC Directive of the European Parliament and of the Council establishes the airborne concentration of asbestos in excess of 0.1 fibers per cubic centimeter as an 8-hour time-weighted average (TWA).^11^ For several years, this exposure limit has been adopted, with some variations, by several European countries.^12,13^

Although the exposure limit values have been lowered over the years, coinciding with the advances in scientific evidence on the harmful effects of asbestos, widespread social debate continues on the existence of a minimum level of intensity of exposure to asbestos below which exposure is safe and above which there is a likelihood of developing health damage. For that reason, continually reviewing and updating information on the health-effects exposure levels of asbestos is important. The aim of this study is to contribute to this debate, reviewing the best and the most recent scientific evidence available in the international literature on exposure levels for asbestos fibers and their relationship with established asbestos health effects.

## Method

An umbrella review^14^ was performed, retrieving systematic reviews and meta- analyses available in MEDLINE/PubMed, Google Scholar Academics bibliographic repository, the International Agency for Research on Cancer (IARC),^3^ the U.S. Agency for Toxic Substances and Disease Registry (ATSDR),^15^ the U.S. Environmental Protection Agency (EPA),^16^ the U.K. Health and Safety Executive (HSE)^17^ and the Canadian Institute for Work and Health (IWH).^18^

Several search strategies were tested using the usual connectors for keywords (AND, OR, NOT, etc.), obtaining final search engines to capture as many references as possible, filtered by “review AND systematic review AND meta-analysis”. The search was restricted to articles published in English from 1980 to March 2021. We included both human and experimental studies in animals.

An initial search strategy was carried out with a wide perspective and using search engines to capture all articles that analyzed the association between asbestos exposure and any known asbestos disease, including lung cancer, mesothelioma, asbestosis, diffuse interstitial pulmonary fibrosis, pleural plaques, diffuse pleural fibrosis, larynx and gastrointestinal cancer. After-wards, different key words were incorporated to the search engines to restrict them to those articles that also incorporated variables that quantified exposure (fiber concentration, dose-exposure, exposure-response or dose-response). The syntaxes used in this review are shown in Supplemental text (syntaxes) in online Appendix.

Each of these syntaxes was applied independently and duplicate studies were eliminated. Titles and abstracts were screened using independent peer-review. A third expert resolved discrepancies and independently decided final inclusion for full text analysis. Only systematic reviews and meta-analyses evaluating exposure to asbestos fibers and its relationship to health effects were included in this umbrella review.

A first selection was based on reading the title, including those with the words “asbestos” and “pathology”, excluding those that were not of interest for the purpose of the study or were doubtful. The second selection was based on reading the abstracts, excluding those studies that did not analyze associations between asbestos exposure and the selected pathologies, or were not systematic reviews or meta- analyses. In a third phase, full texts were screened and articles that did not refer to the purpose of the study were excluded.

In a final stage, an evaluation of the methodological quality of the selected studies was performed by means of the AMSTAR (Assessment of Multiple SysTemAtic Reviews) instrument.^19^ The instrument is a reliable and valid measure for the evaluation of the methodological quality of systematic reviews and has proven good face and content validity.^20^ To report the results of this review, the evidence-based set of items for reporting systematic reviews stated by the Preferred Reporting Items for Systematic Reviews and Meta-Analyses (PRISMA) have been followed.^21^

This study has been registered at PROSPERO (https://www.crd.york.ac.uk/prospero/display_record.php?RecordID=185349).

## Results

### Selection of articles

**Figure 1.**
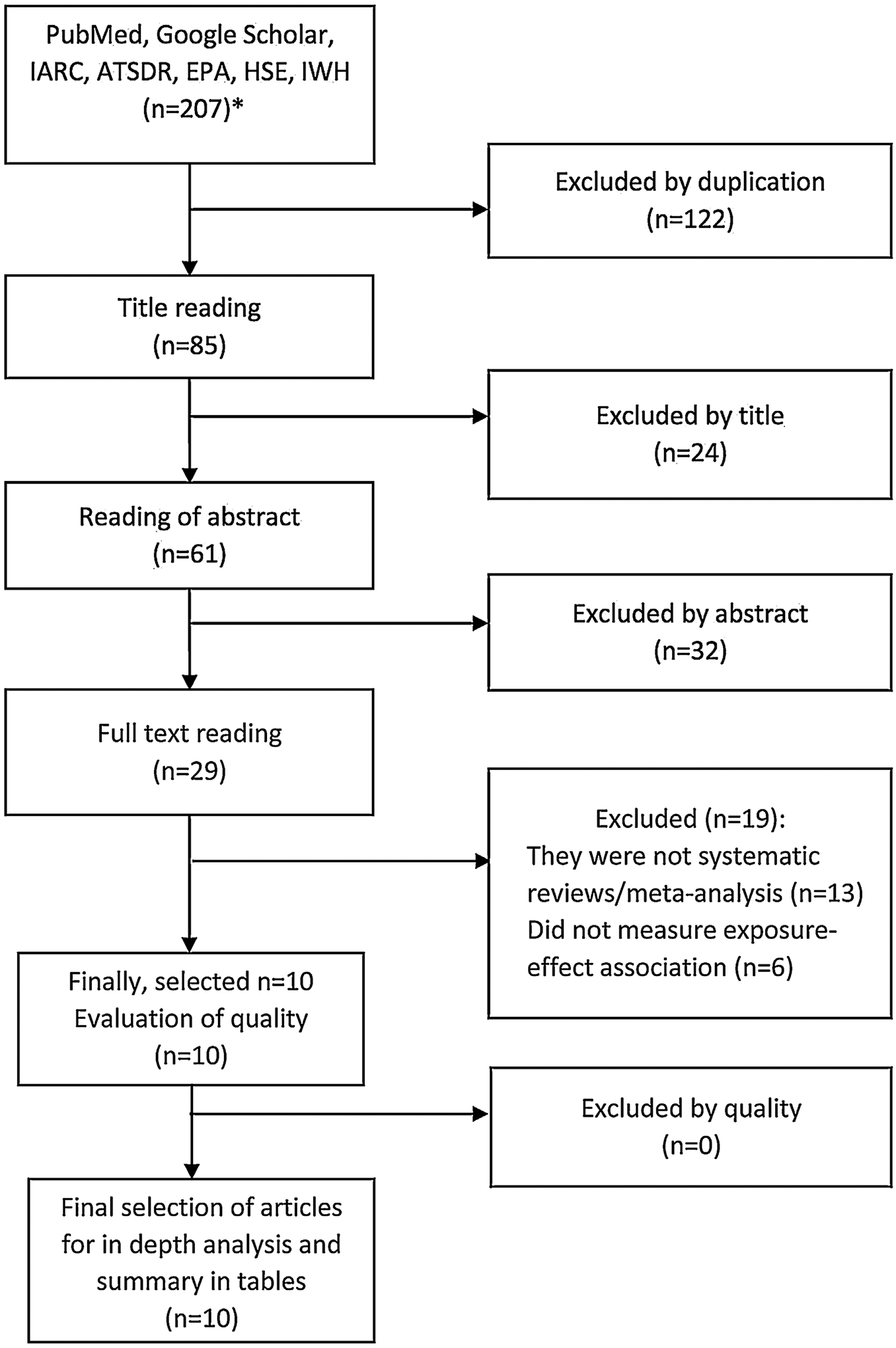
Article selection flowchart (1980–2021). *Referenced in tables in online Appendix. ATSDR: Agency for Toxic Substances and Disease Registry; EPA: U.S. Environmental Protection Agency; HSE: Health and Safety Executive; IARC: International Agency for Research on Cancer; IWH: Canadian Institute for Work and Health.

Initially, 207 references were retrieved (170 from PubMed/MEDLINE and 37 from international agencies), and after removing duplicates (n = 122), there were 85 articles left. Of these, 24 articles were excluded after reading the title and 32 after reading the abstract, obtaining 29 articles. After reading the full text of these studies, 13 were excluded because they were not systematic reviews or meta-analysis and six because they did not have measures of exposure-effect association, obtaining a total of 10 articles for in-depth analysis (fig. 1 and tables II, III and IV in online Appendix).

After evaluating their methodological quality with the AMSTAR instrument, two of them obtained a global score of 10, one a score of 8 and the rest of the studies obtained global scores varying from four to six (table I in online Appendix). However, none of these last 10 articles was excluded, because they provided valuable information (Table 1).

### Characteristics of the studies

The main characteristics of the selected articles are listed in Table 1. Articles included^22–31^ were published between 1997 and 2017, but most (80%) were issued from 2008 onward. Most of the studies (n = 7) were meta-analyses published by researchers from institutions mainly in the U.S., Canada and the Netherlands.^22–25,28,29,31^ One of the reviews^31^ incorporates a multicenter case-control study^32^ with participation from several countries. The reviews and meta-analyses included cohort and case-control studies of reasonably good or very good quality. One of the reviews included is based on experimental animal studies.^30^

Nine systematic reviews/meta-analyses assessed the association between asbestos and lung cancer,^22–30^ six between asbestos and pleural mesothelioma,^23,25–27,30,31^ one between asbestos and pulmonary fibrosis,^30^ and one between asbestos and other gastrointestinal cancers.^27^

### Association between asbestos exposure and lung cancer by fiber type

There was not a clear pattern in the association measures used to analyze the effects, which included the standardized mortality ratio (SMR), relative risk (RR), odds ratio (OR) and percentage of expected mortality per fiber/ml-year of exposure (RL) (Tables 2 and 3). In general, we observed that the effect of chrysotile on lung cancer is characterized by being weaker than the effect of amphibole fibers or mixed fibers.^22–29^ Specifically, the highest risk for lung cancer was observed with exposure to amphibole fibers, followed by mixed-fibers and finally chrysotile.^31^

The length and durability of the fibers was associated with the carcinogenicity potency. Fibers with a length > 10 μm are more carcinogenic than those < 10 μm in length, and chrysotile has been found to be less carcinogenic than amphibole fibers.^25^ The thinner the fiber, the higher its capacity to cause lung cancer, as the fiber can better penetrate the lung tissue. In terms of exposure time, only one study^23^ showed that exposure to 2 or more years to amosite fibers had a higher SMR (SMR = 11.7) than exposure to less than one month (SMR = 2.64). However, one of the studies^26^ concluded that the highest cumulative exposure to chrysotile without an effect of lung cancer is 25 f/ml-y, which means that at this concentration of chrysotile fibers, lung cancer has not yet been observed.

### Association between asbestos exposure and pleural mesothelioma by fiber type

In general, we observed some inconsistency in the results from the different studies regarding exposure-response (Tables 4 and 5). In one study,^23^ the proportionality in the expected mortality risk for mesothelioma was 1:100:500 due to exposures to chrysotile, amosite and crocidolite, respectively. In the same study, the dose-response correlation for amphibole fibers suggested a nonlinear relationship for pleural mesothelioma, and that short-term exposures to high fiber concentrations were at higher risk than exposure to low fiber concentrations with long exposure time.

According to the highest cumulative exposure level at which no effect was observed (NOAEL), results evaluating the exposure-response among the studies were also inconsistent. Two cohorts included in the review by Pierce et al.^26^ did not observe increased risk with the highest cumulative exposures: NOAEL > 400 and ≥ 112 f/cc-y (latency 20 years); and two cohorts included in the same review^26^ observed a NOAEL risk for mesothelioma at 800–1599 f/cc-y and < 15 f/cc-y.

One of the studies^26^ concluded that the highest level of cumulative exposure to chrysotile without the effect of mesothelioma is 15 f/ml-y, meaning that at this concentration of chrysotile fibers no risk of mesothelioma was observed. In addition, the meta-analysis with the highest methodological quality^31^ concludes that little risk of mesothelioma would be observed with asbestos exposure below 0.1 f/ml.

It is noteworthy that in general, longer and thinner fibers are more pathogenic than short ones, in particular amphiboles ≥5 μm have been associated with increased mesothelioma RR and OR.^25,30^ One study examined non-occupational exposures.^31^ That study showed that amphibole fibers were those with the highest capacity to produce mesothelioma since exposure to amphibole fibers showed a meta-RR 2.5–3.2 times greater than exposure to mixed fibers, and a meta-RR 5.3–5.6 times greater than exposure to pure chrysotile fibers.

### Association between asbestos exposure and pulmonary fibrosis by fiber type

Table 6 shows the main results of the association between asbestos exposure and the development of pulmonary fibrosis and other types of cancer by type of asbestos fiber. One of the studies,^30^ which reviewed experimental animal articles, concluded that the length and durability of the fibers is the factor most associated with the potency of carcinogenicity. Long fibers (>5 μm) are associated with pulmonary fibrosis (asbestosis) and cancer (mesothelioma and lung cancer), with no evidence of pathogenicity for pulmonary fibrosis when exposed to fibers with a length ≤5 μm.

### Association between asbestos exposure and other cancers by fiber type

The study by Gamble^27^ (Table 6) found that the association between gastrointestinal cancer and asbestos exposure does not exist or it is very low (RR, OR, and SMR around 1). The only association with gastric cancer was observed with very high exposures (>140 f/ml-y) to chrysotile (SMR = 1.43) and to amosite (SMR = 3.21) at exposures > 1000 mppcf-y). The same would be true for colon cancer, with an association found at exposures > 140 f/ml-y (SMR = 2.31). The studies included in this review do not provide information about exposure levels of asbestos and other cancers, such as larynx or ovarian cancers.

### Association between asbestos exposure and other asbestos related pathologies

Information on exposure levels of asbestos associated with other well-established pathologies (such as pleural plaques, pleural thickening, diffuse pleural fibrosis, effusion and rounded atelectasis) were outside the scope of this review and were not included.

## Discussion

The first finding is that all asbestos fibers have been associated consistently with lung cancer, mesothelioma and pulmonary fibrosis. In relation to laryngeal and ovarian cancer the causality of asbestos has also been demonstrated, but we did not find any systematic reviews or meta-analyses on these two pathologies to be able to draw conclusions about exposure levels. The evidence is, to date, less conclusive for gastrointestinal tumors.^27^

A second finding is that the risk, varies depending on the type of asbestos, the physicochemical characteristics of these fibers, the intensity of exposure and, for some pathologies, co-exposures with other carcinogens, especially tobacco. The studies clearly point that the greatest risk exists with exposure to amphibole fibers, followed by mixed fibers (amphibole and chrysotile), and finally, chrysotile. However, when analyzing the risk that occurs depending on the intensity of asbestos exposure, some studies suggest that for mesothelioma and lung cancer there may be a threshold below which there is no significantly increased risk of suffering the pathology, whereas other studies cannot conclude the existence of a threshold. What seems quite evident is that the exposure intensity required to produce mesothelioma may be lower than for lung cancer or pulmonary fibrosis (asbestosis).

The joint assessment of the analyzed systematic reviews and meta-analyses leads us to the conclusion that there is little risk of lung cancer or mesothelioma at daily exposure levels below 0.1 f/ml (daily environmental exposure limit value). However, most times the measures are reconstructions made decades after the exposure has taken place, which can have a large margin of error when applied to individual patients.^33^ Our findings could be explained at least in part by the carcinogenetic inflammatory mechanisms of asbestos. As a recent review has shown,^34^ asbestos and other fibers remain in the affected tissue for months to years, triggering a chronic inflammatory process and consequent release of high mobility group protein B1 and other cytokines that maintain this process which may ensue in cancer. This pathogenic mechanism could support the fact that low amounts of fibers, probably below 0.1 f/ml, are less likely to elicit a chronic inflammatory process. Other naturally occurring fibers are present in the environment and some of them (e.g. erionite) are as or more carcinogenic than asbestos.^34,35^

In any case, the most recent cutting-edge research shows that “safe” levels could vary greatly from one individual to another depending on the genetics of each individual. In this regard, for example, heritable mutations of the germline BAP1 and other tumor suppressor genes have been reported to increase susceptibility to asbestos carcinogenesis. It is estimated that 12% of mesotheliomas occur in carriers of these mutations.^36,37^

The occupational exposure limit values proposed by internationally renowned agencies have been drastically reduced over time. The ACGIH has adopted the current TLV of 0.1 f/ml for all types of asbestos.^38^ The limit values proposed by other agencies such as OSHA,^10^ NIOSH,^39^ CCOHS,^40^ HSE^41^ and UE-OSHA^42^ currently set an exposure limit value for eight-hour time-weighted average of 0.1 f/ml (with certain particularities). However, as both the ILO and the WHO have stated, there is no safe level of exposure to a carcinogen and, in concordance with them, we advocate for the global ban and eradication of all types of asbestos and demand the complete elimination of asbestos-related diseases as a global public health priority.^43,44^ This review, analyzed and synthesized the main results of 10 systematic reviews and available meta-analyses of the last 40 years that have examined asbestos exposure values that pose a threat to human health. Likewise, the fact that the first article included in the review was published in 1997 and the last in 2017 ensures that our review of reviews included all the reviews and meta-analyses published over the last 40 years.

None of the selected reviews scored low in the assessment of their methodological quality; some of them obtained intermediate quality scores (4 ≥ P ≤ 5) [22–27,30] and 50% of the reviews obtained middle^25,26^ or high quality scores (P ≥ 8),^28,29,31^ supporting a reasonably good quality of the included studies.

As any scientific study this one may have some limitations. It is possible in this type of studies, based on systematic search for scientific publications, to overlook unpublished studies (publication bias). Nevertheless, the fact that we included in our search those studies available on the websites of the leading institutions world-wide which have evaluated research on the health consequences of asbestos exposure, including IARC (WHO), NIOSH (USA), ACGIH (USA), ATSDR (USA), EPA (USA), IWH (Canada) and HSE (UK), it is unlikely that we have left out any major review or meta- analyses performed in recent years.

## Conclusion

In summary, several studies provide a value for asbestos exposure below which no risk of lung cancer or mesothelioma would be observed^26,28,31^ and scientific evidence reported in the highest quality reviews and meta-analyses identified in this umbrella review (those with a 10 on the quality assessment), support that there is only limited evidence of the risk of lung cancer or mesothelioma at daily exposure levels below 0.1 f/ml (daily exposure environmental limit value). However, following the recommendations of the ILO and WHO, the best measure to prevent the negative health effects of asbestos exposure is its banning and eradication following strict cleaning protocols.^43,44^

## Supplementary Material

Supplemental Table 4

Supplemental Table 3

Supplemental Table 5

Supplemental Table 1

Supplemental Table 2

## Figures and Tables

**Table 1:** Main characteristics of the selected articles and exposure data (1980–2021).

Author, year (country)	Type of study	N° of studies included in the analyses	Pathology	Type of fiber	Exposure measure^a^	Methodological quality AMSTAR^b^
Lash T et al., 1997 (USA)	Meta-analysis	15 cohort studies	Lung cancer	Chrysotile	<100 to >400 f-y/ml; <1 to ≥1000 mppcf-y	4
				Amphibole: crocidolite Amphibole: amosite Amphibole: tremolite	<10 to >100 f-y/ml <6 to ≥250 f-y/ml; <1 month & ≥ 2 years exposure <25 to >500 f-y/ml	
				Mixed	<2.7 to ≥150 f-y/ml; <6 to ≥750 mppcf-y	
Hodgson J et al., 2000 (UK)	Meta-analysis	17 cohort studies	Lung cancer Mesothelioma	Chrysotile	22 to 600 f/ml-y	5
				Amphibole: crocidolite Amphibole: amosite	16.4 to 120 f/ml-y 23.6 to 65 f/ml-y	
				Mixed	13 to 750 f/ml-y	
Berry G et al., 2008 (Canada)	Overview	2 meta-analysis (11 cohorts in common)	Lung cancer	Chrysotile	25 f/ml-y	4
				Amphibole: crocidolite Amphibole: amosite	No exposure measures	
				Mixed	No exposure measures	
Berman DW et al., 2008 (USA)	Meta-analysis	15 cohort studies	Lung cancer Mesothelioma	Chrysotile	Length >10 μm; thickness <0.2; <0.4 and >0.2 μm. Not concentrations measures	6
				Amphibole	Length >10 μm; thickness <0.2; <0.4 and >0.2 μm. No concentrations measures	
Pierce JS et al., 2008 (USA)	Review	14 cohort studies	Lung cancer Mesothelioma	Chrysotile	1.4–2.7 to 1600–3200 f/ml-y	6
Gamble JF, 2008 (USA)	Review	43 studies (cohorts and case-control)	Lung cancer Mesothelioma Gastric cancer Colon & rectum cancer	Chrysotile	1 a 356 f/ml-y; <3 to >1000 mppcf-y	5
				Amphibole: amosite	<6 to >250 f/ml-y	
				Mixed	<10 and >10 years exposed	
Lenters V et al., 2011 (The Netherlands)	Meta-analysis	19 studies (18 cohorts and 1 case-control)	Lung cancer	Chrysotile	100 f-y/ml	10
				Amphibole	100 f-y/ml	
				Mixed	100 f-y/ml	
Van der Bij S et al., 2013 (The Netherlands)	Meta-analysis	19 studies (17 cohorts and 2 case-control)	Lung cancer	Chrysotile	4–40 f-y/ml	8
				Amphibole	4–40 f-y/ml	
				Mixed	4–40 f-y/ml	
Roggli V, 2015 (USA)	Review	11 studies (3 case-control, 10 experimental in animals)	Lung cancer Mesothelioma Alveolitis Pulmonary fibrosis	Chrysotile	Length >5 μm. No concentrations measures	4
				Amphibole: crocidolite	Length ≥8 and 10 μm No concentrations measures	
Marsh G et al., 2017 (USA)	Review and Meta-analysis	18 studies (4 ecologic, 10 case-control, 4 cohorts)	Pleural mesothelioma	Chrysotile	No exposure measures	10
				Amphibole: crocidolite	20 f/ml-y	
				Mixed	10–24.2 f/ml-y	

a Exposure values from which the risk will be evaluated. The minimum and maximum range, if any, are detailed. Fiber per milliliter and year (f/ml-y); fibers per year and milliliter (f-y/ml); millions of particles per cubic foot per year (mppcf-y); micrometers (μm); The unit fiber-year milliliter (f-y/ml) and fibers milliliter-year (f/ml-y) are equivalent.

b AMSTAR: assessment of multiple systematic reviews, is a tool to evaluate the methodological quality of the reviews. Result 1 to 11 shows worse and better quality respectively.

**Table 2:** Main results on lung cancer from the selected studies.

Author, year	Type of fiber	Exposure^a^ Min.range; Max. range	Main effect results^b^ (IC95%)	Other observations and results
Lash T et al., 1997	Chrysotile	<100 f-y/ml; >400 f-y/ml	SMR = 0.8 (0.21–2.01); SMR = 1.1 (0.55–2.11)	Measurement of the power of asbestos to cause lung cancer by the cumulative exposure coefficient of asbestos (K1). Given the fixed effects model: K1 = 0.42×10^−3^ (0.22–0.69×10^−3^) ml/f-y. Given the random effects model: K1 = 2.6×10^−3^ (0.65–7.4×10^−3^)ml/f-y. For mining cohorts: K1 = 0.25×10^−3^ (0.01–0.45×10^−3^) ml/f-y. For cement production cohorts: K1 = 3.4×10^−3^ (0.1–8.8×10^−3^) ml/f-y. For textile cohorts: K1 = 7.7×10^−3^ (4.7–12×10^−3^) ml/f-y.
		<1 mppcf-y; ≥1000 mppcf-y	SMR = 1.17 (0.71–1.83); SMR = 3.04 (1.9–4.6)	
	Amphibole: crocidolite Amphibole: amosite	28.8 f-y/ml; Value not given value	SMR = 2.64 (2.15–3.24) SMR = 2.64 (1.44–4.42); SMR = 11.7 (5.83–20.94)	
	Amphibole: tremolite	<1 month exposure; ≥2 years exposure	SMR = 2.06 (0.41–6.0); SMR = 7.91 (5.47–11.05)	
			SMR = 2.04 (0.82–4.21); SMR = 5.58 (1.49–14.22)	
		<6 f-y/ml; ≥250 f-y/ml		
		<25 f-y/ml; >500 f-y/ml		
	Mixed	<2,7 f-y/ml; >150f-y/ml	SMR = 1.4 (0.42–3.07); SMR = 2.69 (0.3–9.71)	
		<6 mppcf-y; ≥750 mppcf-y	SMR = 1.04 (0.21–3.02); SMR = 7.78 (3.12–16.03)	
Hodgson J et al., 2000	Chrysotile	22 f/ml-y; 600 f/ml-y	R_L_ = 1.3 (−0.29–3.4); R_L_ = 0.06 (0.042–0.079)	Measurement of lung cancer risk calculated as expected percentage of mortality per fiber/ml-year of exposure: RL = 100 (O-E) / E RL in exposed to chrysotile = 0.062 (p<0.001); to crocidolite RL = 4.2 (IC95% 2.8–5.8) p = 0.09; to amosite RL = 5.2 (IC95% 4.0–6.5) p = 0.022; and to mixed fibers RL = 0.47 (p<0.001). Long fibers represent an increased risk of carcinogenicity. Short exposure times at high exposure results in a higher risk than long exposure times at low concentration.
	Amphibole: crocidolite	16.4 f/ml-y; 120 f/ml-y	R_L_ = 5.2 (0.71–12.0); R_L_ = 10.0 (3.9–21.0)	
	Amphibole: amosite	23.6 f/ml-y; 65 f/ml-y	R_L_ = 1.9 (−0.44–5.1); R_L_ = 5.8 (4.4–0.74)	
	Mixed	13 f/ml-y; 750 f/ml-y	R_L_ = 6.2 (−0.77–21.0); R_L_ = 0.21 (0.14–0.3)	
Berry G et al., 2007	Chrysotile	25 f/ml-y	RR = 2.05; RR = 1.025; RR = 1.06^c^	Higher risk of amphibole exposure was observed. There is no significant increase in lung cancer. Taconite miners: No clear increase in lung cancer mortality 10 years after first exposure.
			R_L_ = 0.062	
	Amphibole: crocidolite	General	R_L_ = 4.2	
	Amphibole: amosite		R_L_ = 5.25%	
	Mixed	General	R_L_ = 0.47	
Berman W et al., 2008	Chrysotile	Length >10 μm and Thickness <0.2;< 0.4; >0.2 μm	K_L_ = 0.38 (0,0–1.3); K_L_ = 0,49 (0.092–1.4); K_L_ = 0,52 (0.13–1.3)	Fibers >10 μm long are more damaging. There is no clear evidence of carcinogenicity in fibers < 10 μm long. For long fibers (>10 μm), chrysotile has lower risk for lung cancer than long fibers of amphibole. As the thickness decreases, the potency for the lung cancer is increased by both amphibole and chrysotile fibers. Amphibole
	Amphibole (general)	Length >10 μm and Thickness <0.2;<0.4;>0.2 μm	K_L_ = 24.5 (7.6–66.3); K_L_ = 7.7 (1.6–26.6); K_L_ = 3.2 (0.71–14.0)	
Pierce JS et al., 2008	Chrysotile	1.4–2.7 f/ml-y	SMR = 0.65 (0.28–1.43)	Most articles do not show an increased risk of lung cancer from high chrysotile exposures. Cross-contamination with other fibers should be considered. There was no increase in risk of lung cancer in the 4 highest exposure categories (≥112 fibers/cc-y), due to a possible confounding factor in smoking. In other studies, the risk increased from exposures >25-1600-3200 fibers/cc-y. in Cross-contamination with other fibers should be considered.
		414–942 f/ml-y	SMR = 1.05 (0.7–1.52)	
			NOAEL for lung cancer: >25f/cc-y	
Gamble J, 2008	Chrysotile	<15 f-y/ml; >40 f-y/ml	RR = 1.8 (0.8–3.9); 1.9 (0.5–7.1)	
		<3 mppcf-y; >1000 mppcf-y	SMR = 1.12; SMR = 2.95 (2.18–3.96)	
	Amphibole: amosite	<6 f/ml-y; >250 f/ml-y	SMR = 2.64; SMR = 11.7	
	Mixed	<10 and >10 years exposed	SMR = 0.92; SMR = 1.41	
Lenters V et al., 2011	Chrysotile	100 f-y/ml	K_L_ = 0.04 (−0.05–0.12)	Applying formula RR = α (1 + K_L_ × CE) where α is the rate for lung cancer with exposure 0, K_L_ is the increment coefficient of RR per unit in fibers-y/ml and CE the accumulated exposures (if available for 10 years) can be obtained if α=1.47, K_L_ =0.13, a metaRR = 1.66 (1.53–1.79) for each100 f-y/ml. RR = 1.013 (0.791–1.296) for 4 f-y/ml (fibers in general) RR = 1.133 (0.888–1.444 for 40 f-y/ml (fibers in general) Linear increase in risk of lung cancer at low levels of exposure. The slope decreases slightly from exposures to 150 f-y/ml. Increased risk of lung cancer from exposure to amphibole fibers, followed by mixed fibers and chrysotile.
	Amphiboles (General)	100 f-y/ml	K_L_ = 0.33 (0.09–0.56)	
	Mixed	100 f-y/ml	K_L_ = 0.13 (0.03–0.23)	
Van der Bij S et al., 2013	Chrysotile	4 f-y/ml −40 f-y/ml	RR = 1.006 (0.848–1.194); RR = 1.064(0.873–1.297)	
	Amphibole (General)	4 f-y/ml −40 f-y/ml	RR = 1.022(0.568–1.837); RR = 1.232(0.687–2.209)	
	Mixed	4 f-y/ml −40 f-y/ml	RR = 1.018(0.690–1.503); RR = 1.194(0.825–1.727)	
Roggli V, 2015	Chrysotile	Length>5 μm,	RR and OR increase (not specified)	No clear evidence of carcinogenicity for amphibole fibers >10 μm and chrysotile ≤5 μm long. The length and durability of the fibers is what is most associated with the potency of carcinogenicity. Other non-asbestiform long fibers have also been associated with fibrosis and tumors.
	Amphibole: crocidolite	Length ≥10 μm	RR and OR increase (not specified)	

a Exposure values from which the risk will be evaluated. The minimum and maximum range, if any, are detailed. The unit fiber-year/milliliter (f-y/ml), fibers/milliliter-year (f/ml-y), fibers-year/cubic centimeter (f-y/cc) and fibers/cubic centimeter-year (f/cc-y) are equivalent; General: fibers without exact measurement of exposure.

b R_L_: risk of lung cancer; excess risk of lung cancer risk per fiber/ml-year; equivalent to KL; RL = 100 (SMR-1)/Expos (f/ml-y).

c Different RRs depending on the applied coefficient: following EPA models (RR = 1 + 0.01 cumulative exposure), Quebec mines (RR = 1 + 0.0006 accumulated exposure), industry (RR = 1 + 0.00025 accumulated exposure). The latter would be most appropriate for the indicated study. NOAEL: highest cumulative exposure level at which no effect was observed; RR: relative risk; OR: odds ratio; SMR: standardized mortality ratio. Some results have no confidence interval (95% CI).

**Table 3:** Main results on lung cancer according to fiber type.

Fiber type		Exposure^a^ Min. range; Max. range	Main effect results^b^ (IC95%)	Author, year
Chrysotile		<100 f-y/ml; >400 f-y/ml	SMR = 0.8 (0.21–2.01); SMR = 1.1 (0.55–2.11)	Lash T et al., 1997
		<1 mppcf-y; ≥1000 mppcf-y	SMR = 1.17 (0.71–1.83); SMR = 3.04 (1.9–4.6)	
		22 f/ml-y; 600 f/ml-y	R_L_ = 1.3 (−0.29–3.4); R_L_ = 0.06 (0.042–0.079)	Hodgson J et al., 2000
		25 f/ml-y	RR = 2.05; RR = 1.025; RR = 1.06^c^	Berry G et al., 2007
		Length >10 μm; Thickness	Kl = 0.38 (0.0–1.3); Kl = 0.49 (0.092–1.4);	Berman W et al., 2008
		<0.2;<0.4; >0.2 μm	K_L_ = 0.52 (0.13–1.3)	
		1.4–2.7 f/ml-y; 414–942 f/ml-y	SMR = 0.65 (0.28–1.43); SMR = 1.05 (0.7–1.52)	Pierce JS et al., 2008
		<15 f-y/ml; >40 f-y/ml	RR = 1.8 (0.8–3.9); 1.9 (0.5–7.1)	Gamble J, 2008
		<3 mppcf-y; >1000 mppcf-y	SMR = 1.12; SMR = 2.95 (2.18–3.96)	
		100 f-y/ml	K_L_ = 0.04 (−0.05–0.12)	Lenters V et al., 2011
		4 f-y/ml −40 f-y/ml	RR = 1.006 (0.848–1.194);	Van der Bij S et al., 2013
			RR = 1.064(0.873–1.297)	
		Length >5 μm.	RR and OR increase (not specified)	Roggli V, 2015
Amphibole	Crocidolite	<10 f-y/ml; >100 f-y/ml	SMR = 2.64 (2.15–3.24); SMR = 2.64 (2.15–3.24)	Lash T et al., 1997
		16.4 f/ml-y; 120 f/ml-y	R_L_ = 5.2 (0.71–12.0); R_L_ = 10.0 (3.9–21.0)	Hodgson J. et al., 2000
		General	R_L_ = 4.2	Berry G et al., 2007
	Amosite	<1 month exposure; ≥2 years exposure	SMR = 2.64 (1.44–4.42); SMR = 11.7 (5.83–20.94)	Lash T et al., 1997
		<6 f-y/ml; >250 f-y/ml	SMR = 2.06 (0.41–6.0); SMR = 7.91 (5.47–11.05)	
		23.6 f/ml-y; 65 f/ml-y	R_L_ = 1.9 (−0.44–5.1); R_L_ = 5.8 (4.4–0.74)	Hodgson J et al., 2000
		General	R_L_ = 5.2	Berry G et al., 2007
		<6 f/ml-y; >250 f/ml-y	SMR = 2.64; SMR = 11.7	Gamble J. 2008
	Tremolite Amphibole in general	<25 f-y/ml; >500 f-y/ml	SMR = 2.04 (0.82–4.21); SMR = 5.58 (1.49–14.22)	Lash T et al., 1997
		Length >10 μm; Thickness <0.2;<0.4; >0.2 μm	K_L_ = 24.5 (7.6–66.3); K_L_ = 7.7 (1.6–26.6); K_L_ = 3.2 (0.71–14.0)	Berman W et al., 2008
		100 f-y/ml	K_L_ = 0.33 (0.09–0.56)	Lenters Vet al., 2011
		4 f-y/ml −40 f-y/ml	RR = 1.022(0.568–1.837); RR = 1.232(0.687–2.209)	Van der Bij S et al., 2013
		Length ≥10 μm	RR and OR increase (not specified)	Roggli V, 2015
Mixed		<2.7 f-y/ml; >150f-y/ml	SMR = 1.4 (0.42–3.07); SMR = 2.69 (0.3–9.71)	Lash T et al., 1997
		<6 mppcf-y; ≥750 mppcf-y	SMR = 1.04 (0.21–3.02); SMR = 7.78 (3.12–16.03)	
		13 f/ml-y; 750 f/ml-y	R_L_ = 6.2 (−0.77–21.0); R_L_ = 0.21 (0.14–0.3)	Hodgson J et al., 2000
		General	R_L_ = 0.47SMR = 1.17 (0.71–1.83); SMR = 3.04 (1.9–4.6)	Berry G et al., 2007
		<10 years exposed; >10 years exposed	SMR = 0.92; SMR = 1.41	Gamble J, 2008
		100 f-y/ml	K_L_ = 0.13 (0.03. 0.23)	Lenters V et al., 2011
		4 f-y/ml −40 f-y/ml	RR = 1.018 (0.690–1.503); RR = 1.194 (0.825–1.727)	Van der Bij S et al., 2013

a Exposure values from which the risk will be evaluated. The minimum and maximum range, if any, are detailed. The unit fiber-year/milliliter (f-y/ml), fibers/milliliter-year (f/ml-y), fibers-year/cubic centimeter (f-y/cc) and fibers/cubic centimeter-year (f/cc-y) are equivalent; General: fibers without exact measurement of exposure.

b R_L_: risk of lung cancer; excess risk of lung cancer risk per fiber/ml-year; equivalent to KL; RL = 100 (SMR-1)/Expos (f/ml-y).

c Different RRs depending on the applied coefficient: following EPA models (RR = 1 + 0.01 cumulative exposure), Quebec mines (RR = 1 + 0.0006 accumulated exposure), industry (RR = 1 + 0.00025 accumulated exposure). The latter would be most appropriate for the indicated study. NOAEL: highest cumulative exposure level at which no effect was observed; RR: relative risk; OR: odds ratio; SMR: standardized mortality ratio. Some results have no confidence interval (95% CI).

**Table 4:** Main results on pleural mesothelioma from the selected studies.

Author, year	Type of fiber	Exposure^a^ Min. range; Max. range	Main effect results^b^ (IC95%)	Other observations and results
Hodgson J et al., 2000	Chrysotile	22 f/ml-y; 600 f/ml-y	R_M_ = 0.0 (0.0–0.033); R_M_ = 0.00096 (0.0006–0.0013)	Expected mortality risk for mesothelioma is 1:100:500 due to exposures to chrysotile, amosite and crocidolite, respectively. Expected mortality per fiber/ml-year in crocidolite exposed = 0.51 (95% CI = 0.1–0.61) p = 0.6; to amosite = 0.10 (95% CI = 0.062–0.15) p = 0.2; to chrysotile = 0.0010 (95% CI = 0.0007–0.00014) p = 0.11 and to mixed fibers = 0.021 (p < 0.001).
	Amphibole: crocidolite Amphibole: amosite	16.4 f/ml-y; 120 f/ml-y 23.6 f/ml-y; 65 f/ml-y	R_M_ = 0.59 (0.36–0.91); R_M_ = 0.68 (0.22–1.6) R_M_ = 0.06 (0.016–0.015); R_M_ = 0.12 (0.068–0.19)	
	Mixed	13 f/ml-y; 750 f/ml-y	R_M_ = 0.2 (0.11–0.35); R_M_ = 0.001 (0.0005–0.0028)	
Berman W et al., 2008	Chrysotile	Length >10 μm and Thickness <0.2;<0.4; >0.2 μm	K_M_ = 0.0 (0.0–0.27); K_M_ = 0.0 (0.0–0.34); K_M_ = 0.01 (0.0–0.31)	It is rejected the hypothesis that chrysotile and amphibole fibers are equally powerful. Chrysotile is less powerful than amphiboles in causing mesothelioma. Chrysotile is less potent than amphiboles in causing mesothelioma. It has almost zero power. Fibers >10 μm in length are more powerful than short ones.
	Amphiboles (global)	Length >10 μm and Thickness <0.2;<0.4; >0.2 μm	K_M_ = 32.0 (0.0–89.9); Km = 30.8 (16.5–61.5); K_M_ = 19.9 (8.8–41.2)	
Pierce JS et al., 2008	Chrysotile	<15 f/ml-y; 800–1599 f/ml-a	RR = 1.9 (0.2–21.3); RR = 0. NOAEL for mesothelioma <15f/ml-y	Cross-contamination with other fibers should be monitored. Difficulty identifying and cataloging cumulative exposure below which there is no increase in mesothelioma risk. Inconsistency of the results given by the studies when evaluating the exposure-response. Two cohorts did not observe increased risk with the highest cumulative exposures. NOAEL >400 (Piolatto et al., 1990) and ≥112 (McDonald et al., 1984) f/cc-y (latency 20 years). Two cohorts observe a NOAEL risk for mesothelioma of 800–1599 f/cc-y (Lacquet et al., 1980) and < 15 f/cc-y (Albin et al., 1990).
Gamble J, 2008	Chrysotile	<15 f-y/ml; >40 f-y/ml	SMR = 1.9 (0.2–21.0); SMR = 23 (2.4–212.0)	
Roggli V, 2015	Amphibole: crocidolite. amosite and tremolite	Length ≥5 and 10 μm	RR and OR increase (not specified)	According to Dodson et al. the only fibers found in the pleural tissue are fibers >10 μm long. The length and durability of the fairs is what is most associated with the potency of carcinogenicity. Long fibers (>5 μm) are most associated with mesothelioma. McDonald et al. (1989) show risk associated with amphibole fibers ≥8 μm. Rödelsperger et al. (1999) to amphibole fibers >5 μm and Rogers et al. (1991) with crocidolite fibers ≥10 μm. Other non-asbestiform long fibers have also been associated with fibrosis and tumors.
Marsh G et al., 2017	Amphibole: crocidolite	20 f/ml-y	RR = 3.6 (IC95% 1.3–9.5) and RR = 6.3 (IC95% 1.3–9.5)	Non-occupational exposures, at home and residential level. Exposures to residential chrysotile: OR = 0.2 (0.02–2.2) Exposures to residential mixed fibers: SMR = 87.0 (32.5–233) MetaRR at the residential level vs. Domestic: Chrysotile: 3.8 (0.4–38.3) vs. 4.0 (0.8–18.8); Mixed: 8.4 (4.7–14.9) vs. 5.3 (1.9–15.0); Amphiboles: 21.1 (5.3–84.5) vs. 21.1 (2.8–156.0). Mixed
	Mixed	10–24.2 f/ml-y	OR = 23.3 (2.9–186.9)	

a Exposure values from which the risk will be evaluated. The minimum and maximum range, if any, are detailed. The units fiber-year/milliliter (f-y/ml), fibers/milliliter-year (f/ml-y), fibers-year/cubic centimeter, (f-y/cc) and fibers/cubic centimeter-year (f/cc-y) are equivalent.

b R_M_: risk of mesothelioma¸ excess mesothelioma mortality per fiber/ml-year; equivalent to K_M_; NOAEL: highest cumulative exposure level at which no effect was observed; RR: relative risk; OR: odds ratio; SMR: standardized mortality ratio. Some results have no confidence interval (95% CI).

**Table 5:** Main results obtained for pleural mesothelioma according to fiber type.

Type of fiber		Exposure^a^ Min. range; Max. range	Main effect results^b^ (IC95%)	Author, year
Chrysotile		22 f/ml-y; 600 f/ml-y	R_M_ = 0.0 (0.0–0.033); R_M_ = 0.00096 (0.0006–0.0013)	Hodgson J et al., 2000
		Length >10 μm and thickness <0.2;<0.4; >0.2 μm	K_M_ = 0.0 (0.0–0.27); K_M_ = 0.0 (0.0–0.34); K_M_ = 0.01 (0.0–0.31)	Berman W et al., 2008
		<15 f/ml-y; 800–1599 f/ml-y	RR = 1.9 (0.2–21.3); RR = 0. NOAEL for mesothelioma <15 f/ml-y	Pierce JS et al., 2008
		<15 f-y/ml; >40 f-y/ml	SMR = 1.9 (0.2–21.0); SMR = 23 (2.4–212.0)	Gamble J, 2008
Amphibole	Crocidolite	16.4 f/ml-y; 120 f/ml-y	R_M_ = 0.59 (0.36–0.91); R_M_ = 0.68 (0.22–1.6)	Hodgson J et al., 2000
		20 f/ml-y	RR = 3.6–6.3	Marsh. G et al., 2017
	Amosite General	23.6 f/ml-y; 65 f/ml-y	R_M_ = 0.06 (0.016–0.015); R_M_ = 0.12 (0.068–0.19)	Hodgson et al., 2000
		Length >10 μm and thickness	K_M_ = 32.0 (0.0–89.9); K_M_ = 30.8	Berman W et al., 2008
		<0.2;<0.4; >0.2 μm	(16.5–61.5); K_M_ = 19.9 (8.8–41.2)	
		Length ≥5 and 10 μm	RR and OR increase (not specified)	Roggli V, 2015
Mixed		13 f/ml-y; 750 f/ml-y	R_M_ = 0.2 (0.11–0.35); R_M_ = 0.001 (0.0005–0.0028)	Hodgson J et al., 2000

a Exposure values from which the risk will be evaluated. The minimum and maximum range, if any, are detailed. The units fiber-year/milliliter (f-y/ml), fibers/milliliter-year (f/ml-y), fibers-year/cubic centimeter (f-y/cc) and fibers/cubic centimeter-year (f/cc-y) are equivalent.

b R_M_: risk of mesothelioma¸ excess mesothelioma mortality per fiber/ml-year; equivalent to K_M_; NOAEL: highest cumulative exposure level at which no effect was observed; RR: relative risk; OR: odds ratio; SMR: standardized mortality ratio. Some results have no confidence interval (95% CI).

**Table 6:** Main results on pulmonary fibrosis and other cancers from the selected studies, according to fiber type.

Author, year	Type of fiber	Exposure^a^ Min. range; Max. range	Main effect results^b^ (IC95%)	Other observations and results
Roggli V, 2015	Chrysotile	<8 μm 23 million chrysotile fibers >5 μm in animals and 272 million ≤5 μm with 18–24 months of exposure	Increased risk of alveolitis but not pulmonary fibrosis. No increased risk of fibrosis, RR and OR not specified.	Long fibers (>5 μm) are associated with a higher risk of pulmonary fibrosis (asbestosis) than short ones. There is no evidence of pathogenicity in ≤5 μm fibers.
	Amphibole: crocidolite	Intratracheal injection of short fibers Intratracheal injection of long fibers	Increased risk of minimal lung injury or fibrosis. Increased risk of severe fibrosis (similar to human asbestosis).	Other non-asbestiform long fibers have also been associated with fibrosis and tumors.
Gamble J, 2008	Chrysotile	<15 f-y/ml; >40 f-y/ml 10–49 f/ml-y; 100–356 f/ml-y <8.4 f/ml-y; >140 f/ml-y <300 mppcf-y; >1000 mppcf-y	RR = 1.0; RR = 3.4 (1.2–9.5) for colorectal cancer. RR = 1.0; RR = 1.7 (0.2–3.3) for gastric cancer. OR = 1.18; OR = 0.28 (gastrointestinal cancer). SMR = 1.06 (0.65–1.63); SMR = 2.31 (1.19–4.04) for colon cancer. SMR = 0.51 (0.66–1.85); SMR = 0.0 for colorectal cancer. SMR = 0.96 (0.19–2.7); SMR = 1.43 (0.04–7.96) for gastric cancer. SMR = 0.93 (0.82–1.05); 1.12 (0.63–1.85) for colorectal cancer. SMR = 1.16 (0.96–1.39); SMR 3.21 (1.87–5.13) for gastric cancer.	The association between gastrointestinal cancer and exposure to asbestos is very weak. Only association with gastric cancer was observed with very high exposures. Possible cross-contamination with other fibers should be considered.
	Amphibole: amosite Mixed	<6 f/ml-y; >250 f/ml-y <10 years exposed; >10 years exposed	SMR = 1.66 (0.71–3.26); SMR = 1.96 (0.24–7.08) for gastrointestinal cancer SMR = 0.42; SMR = 0.45 for colon cancer. SMR = 0.52; SMR = 0.82 for rectal cancer. SMR = 1.50; SMR = 0.92 for gastric cancer.	

a Exposure values from which the risk will be evaluated. The minimum and maximum range, if any, are detailed. The units fiber-year/milliliter (f-y/ml), fibers/milliliter-year (f/ml-y), fibers-year/cubic centimeter (f-y/cc) and fibers/cubic centimeter-year (f/cc-y) are equivalent.

b RR: relative risk; OR: odds ratio; SMR: standardized mortality ratio. Some results have no confidence interval (95% CI).

## What is known about the topic?

Although the exposure limit values for asbestos have been lowered over the years, coinciding with the advances in scientific evidence on the harmful effects of asbestos, widespread social debate continues on the existence of a minimum level of intensity of exposure to asbestos below which exposure is safe and above which there is a likelihood of developing health damage.

## What does this study add to the literature?

This study contributes to this debate, reviewing the best and the most recent scientific evidence available in the international literature on exposure levels for asbestos fibers and their relationship with established asbestos health effects.

## What are the implications of the results?

There is limited evidence of the risk of lung cancer or mesothelioma at daily exposure levels below 0.1 f/ml (VLA-ED). However, the best measure to prevent negative health effects of asbestos exposure is its banning and eradication following strict cleaning protocols.

## References

[1] Agudo A, González CA. Exposición al amianto y sus efectos sobre la salud. Arch Prev Riesgos Labor. 2001;2:55–7.

[2] Rego Fernández G, Rego Álvarez R. Enfermedades respiratorias ocupacionales y medioambientales Fundamentos para su investigación clínico-epidemiológica. Asturias: Sociedad Asturiana de Medicina y Seguridad del Trabajo; 2011. p. 153–76.

[3] International Agency for Research on Cancer (IARC). IARC monographs on the identification of carcinogenic hazards to humans. Volume 100C: Asbestos (chrysolite, amosite, crocidolite, tremolite, actinolite and anthophillite). Lyon (France): IARC;; 2012. p. 219–309 [Accessed 2021 April 01]. Available at: https://monographs.iarc.fr/monographs-available/#25.

[4] Artieda L, Beloqui A, Lezaun M. Cohorte poblacional de trabajadores expuestos a amianto Navarra 1999–2004. An Sist Sanit Navar. 2005;28:335–44. 16421611 10.4321/s1137-66272005000500004

[5] Ministerio de Trabajo, Migraciones y Seguridad Social. Instituto Nacional de Seguridad e Higiene en el Trabajo (INSHT). Guía técnica para la evaluación y prevención de los riesgos relacionados con la exposición al amianto. RD 396/2006, BOE n° 86, de 11 de abril. [Accessed 2021 April 01]. Available at: https://cutt.ly/8ryw0z8.

[6] Ministerio de Trabajo y Asuntos Sociales. Instituto Nacional de Seguridad e Higiene en el Trabajo (INSHT). Nota Técnica de Prevención (NTP) 463: Exposición a fibras de amianto en ambientes interiores. 1995 [Accessed 2021 April 01]. Available at: https://cutt.ly/8ryw1G2.

[7] Delclos G, Buffler PA, Greenberg SD, Asbestos-associated diseases: a review. Tex Med. 1989;85:50–9. 2660312

[8] Delclos J, Alarcón M, Casanovas A, Identificación de los riesgos laborales asociados a enfermedad sospechosa de posible origen laboral atendida en el Sistema Nacional de Salud. Aten Primaria. 2012;44:611–27. 22626585 10.1016/j.aprim.2011.11.006 PMC7025942

[9] American Conference of Governmental Industrial Hygienists (ACGIH). TLV/BEI Guidelines. [Updated on 2021]. [Accessed 2021 January 8]. Available at: https://www.acgih.org/tlv-bei-guidelines/policies-procedures-presentations/overview.

[10] Occupational Safety and Health Administration (OSHA). Fact Sheet: Asbestos. 2014 [Accessed 2021 April 01]. Available at: https://www.osha.gov/Publications/OSHA3507.pdf.

[11] Official Journal of the European Union. Directive 2009/148/EC of the European Parliament and of the Council on the protection of workers from the risks related to exposure to asbestos at work. 2009 [Accessed 2021 April 01]. Available at: https://cutt.ly/VrywMuC.

[12] Ministerio de Trabajo, Migraciones y Seguridad Social. Instituto Nacional de Seguridad y Salud en el Trabajo (INSST). Límites de exposición profesional para agentes químicos en España. 2019 [Accessed 2021 April 01]. Available at: https://cutt.ly/BrywB2I.

[13] El Yamani M, Boulanger G, Nerrière-Catelinois E, Revision of French occupational exposure limits of asbestos and recommendation of measurement method: can the dimensional characteristics of the asbestos fibers (long, thin, short) be taken into account? Critical Reviews in Environmental Science and Technology. 2012;42:1441–84.

[14] Hunt H, Pollock A, Campbell P, An introduction to overviews of reviews: planning a relevant research question and objective for an overview. Syst Rev. 2018;7:39. 29490699 10.1186/s13643-018-0695-8 PMC5831229

[15] Agency for Toxic Substances and Disease Registry (ATSDR). Asbestos. [Updated 2011 March 3]. [Accessed 2021 April 01]. Available at: https://www.atsdr.cdc.gov/substances/toxsubstance.asp?toxid=4.

[16] United Estates Environmental Protection Agency (EPA). Asbestos. [Updated 2019 April 16]. [Accessed 2021 April 01]. Available at: https://www.epa.gov/asbestos.

[17] Health and Safety Executive (HSE). Asbestos. [Accessed 2021 April 01]. Available at: http://www.hse.gov.uk/asbestos/information.htm.

[18] Institut for Work and Health. Asbestos. [Updated 2019 December]. [Accessed 2021 April 01]. Available at: https://cutt.ly/LryrzTB.

[19] Shea BJ, Grimshaw JM, Wells GA, Development of AMSTAR: a measurement tool to assess the methodological quality of systematic reviews. BMC Med Res Methodol. 2007;7:10. 17302989 10.1186/1471-2288-7-10 PMC1810543

[20] Shea BJ, Hamel C, Wells GA, AMSTAR is a reliable and valid measurement tool to assess the methodological quality of systematic reviews. J Clin Epidemiol. 2009;62:1013–20. 19230606 10.1016/j.jclinepi.2008.10.009

[21] Moher D, Liberati A, Tetzlaff J, The PRISMA Group Preferred Reporting Items for Systematic Reviews and Meta-Analyses: The PRISMA Statement. PLoS Med. 2009;6:e1000097. 19621072 10.1371/journal.pmed.1000097 PMC2707599

[22] Lash TL, Crouch EA, Green LC. A meta-analysis of the relation between cumulative exposure to asbestos and relative risk of lung cancer. Occup Environ Med. 1997;54:254–63. 9166131 10.1136/oem.54.4.254 PMC1128699

[23] Hodgson J, Darnton A. The quantitative risks of mesothelioma and lung cancer in relation to asbestos exposure. Ann Occup Hyg. 2000;44:565–601. 11108782

[24] Berry G, Gibbs GW. An overview of the risk of lung cancer in relation to exposure to asbestos and of taconite miners. Regul Toxicol Pharmacol. 2008;52 1 Suppl:S218–22. 17998152 10.1016/j.yrtph.2007.09.012

[25] Berman DW, Crump KS. A meta-analysis of asbestos-related cancer risk that addresses fiber size and mineral type. Crit Rev Toxicol. 2008;38 Suppl 1:49–73. 18686078 10.1080/10408440802273156

[26] Pierce JS, McKinley MA, Paustenbach DJ, An evaluation of reported no-effect chrysotile asbestos exposures for lung cancer and mesothelioma. Crit Rev Toxicol. 2008;38:191–214. 18324516 10.1080/10408440701845609

[27] Gamble J Risk of gastrointestinal cancers from inhalation and ingestion of asbestos. Regul Toxicol Pharmacol. 2008;52 1 Suppl:S124–53. 18078700 10.1016/j.yrtph.2007.10.009

[28] Lenters V, Vermeulen R, Dogger S, A meta-analysis of asbestos and lung cancer: is better quality exposure assessment associated with steeper slopes of the exposure-response relationships? Environ Health Perspect. 2011;119:1547–55. 21708512 10.1289/ehp.1002879 PMC3226488

[29] van der Bij S, Koffijberg H, Lenters V, Lung cancer risk at low cumulative asbestos exposure: meta-regression of the exposure-response relationship. Cancer Causes Control. 2013;24:1–12. 23187858 10.1007/s10552-012-0107-7

[30] Roggli VL. The so-called short-fiber controversy: literature review and critical analysis. Arch Pathol Lab Med. 2015;139:1052–7. 26230599 10.5858/arpa.2014-0466-RA

[31] Marsh GM, Riordan AS, Keeton KA, Non-occupational exposure to asbestos and risk of pleural mesothelioma: review and meta-analysis. Occup Environ Med. 2017;74:838–46. 28935666 10.1136/oemed-2017-104383

[32] Magnani C, Agudo A, González CA, Multicentric study on malignant pleural mesothelioma and non-occupational exposure to asbestos. Br J Cancer. 2000;83:104–11. 10883677 10.1054/bjoc.2000.1161 PMC2374531

[33] Takahashi K, Case BW, Dufresne A, Relation between lung asbestos fibre burden and exposure indices based on job history. Occup Environ Med. 1994;51:461–9. 8044245 10.1136/oem.51.7.461 PMC1128015

[34] Carbone M, Adusumilli PS, Alexander A Jr, Mesothelioma: scientific clues for prevention, diagnosis, and therapy. CA Cancer J Clin. 2019;69:402–29. 31283845 10.3322/caac.21572 PMC8192079

[35] Carbone M, Baris YI, Bertino P, Erionite exposure in North Dakota and Turkish villages with mesothelioma. Proc Natl Acad Sci U S A. 2011;108:13618–23. 21788493 10.1073/pnas.1105887108 PMC3158231

[36] Carbone M, Yang H, Pass HI, BAP1 and cancer. Nat Rev Cancer. 2013;13:153–9. 23550303 10.1038/nrc3459 PMC3792854

[37] Carbone M, Arron ST, Beutler B, Tumour predisposition and cancer syndromes as models to study gene–environment interactions. Nat Rev Cancer. 2020;20:533–49. 32472073 10.1038/s41568-020-0265-y PMC8104546

[38] Pira E, Donato F, Maida L, Exposure to asbestos: past, present and future. J Thorac Dis. 2018;10 Suppl 2:S237–45. 29507791 10.21037/jtd.2017.10.126 PMC5830559

[39] Centers for Disease Control and Prevention (CDC). The National Institute for Occupational Safety and Health (NIOSH). Appendix C - Supplementary Exposure Limits. [Accessed 2021 January 8]. Available at: https://www.cdc.gov/niosh/npg/nengapdxc.html.

[40] Canadian Centre for Occupational Health and Safety (CCOHS). Federal government lowers limit of exposure to airborne chrysotile asbestos. [Accessed 2021 January 8]. Available at: https://www.ccohs.ca/oshanswers/chemicals/asbestos/control.html.

[41] Health and Safety executive (HSE). Asbestos – FAQs: what is the control limit? [Accessed 2021 January 8]. Available at: https://www.hse.gov.uk/asbestos/faq.htm.

[42] European Agency for Safety and Health at Work (EU-OSHA). Directive 2009/148/EC-exposure to asbestos at work. [Accessed 2021 January 8]. Available at: https://osha.europa.eu/en/legislation/directives/2009-148-ec-exposure-to-asbestos-at-work.

[43] International Labour Organization (ILO). The ILO Asbestos Convention. 1986;(No. 162). The ILO position on safety in the use of asbestos. Available at: https://www.ilo.org/global/topics/safety-and-health-at-work/areasofwork/occupational-health/WCMS_360580/lang–en/index.htm.

[44] International Labour Organization (ILO). Thirteenth Session of the Joint ILO/WHO Committee on Occupational Health Geneva, 9–12 December 2003. International Labor Office. 2006. Outline for the Development of National Programmes for Elimination of Asbestos-Related Diseases. Available at: https://www.ilo.org/global/topics/safety-and-health-at-work/resources-library/publications/WCMS_108555/lang–en/index.htm.

