# Supplementary material for: Fiber burden and asbestos-related diseases: an umbrella review
Source: Gac Sanit. Author manuscript; Available in PMC 2022 Mar 27. (PMC8882348; doi:10.1016/j.gaceta.2021.04.001)
Supplement: Supplemental Table 4 [file NIHMS1776636-supplement-Supplemental_Table_4.doc]

| Supplementary Table 4. Studies retrieved from other databases (Cochrane, IARC, ATSDR, EPA, HSE and IWH). | |
| --- | --- |
| 1. | Ascoli V, Cavone D, Merler E, Barbieri PG, Romeo L, Nardi F, et al. Mesothelioma in blood related subjects: report of 11 clusters among 1954 Italy cases and review of the literature. Am J Ind Med. 2007;50(5):357-69. |
| 2. | Dement JM, Brown DP. Lung cancer mortality among asbestos textile workers: a review and update. Ann Occup Hyg. 1994;38(4):525-32, 412. |
| 3. | Dodson RF, Atkinson MA, Levin JL. Asbestos fibre length as related to potential pathogenicity: a critical review. Am J Ind Med. 2003;44(3):291-7. |
| 4. | Hagemeyer O, Otten H, Kraus T. Asbestos consumption, asbestos exposure and asbestos-related occupational diseases in Germany. Int Arch Occup Environ Health. 2006;79: 613-20. |
| 5. | Hodgson JT, Darnton A. The quantitative risks of mesothelioma and lung cancer in relation to asbestos exposure. Ann Occup Hyg. 2000; 44: 565–601. |
| 6. | Williams PRD, Phelka AD, Paustenbach DJ. A review of historical exposures to asbestos among skilled craftsmen (1940–2006). J Toxicol Environ Health B Crit Rev. 2007;10(5):319-77. |
| 7. | Lippmann M. Effects of fibre characteristics on lung deposition, retention, and disease. Environ Health Perspect. 1990;88: 311–17. |
| 8. | Loomis D, Dement JM, Richardson D, Wolf S. Asbestos fibre dimensions and lung cancer mortality among workers exposed to chrysotile. Occup Environ Med. 2009;67: 580-84. |
| 9. | Madl AK, Clark K, Paustenbach DJ (2007). Exposure to airborne asbestos during removal and installation of gaskets and packings: a review of published and unpublished studies. J Toxicol Environ Health B Crit Rev. 2007;10(4):259-86. |
| 10. | Lash TL, Crouch EA, Green LC. A meta-analysis of the relation between cumulative exposure to asbestos and relative risk of lung cancer. Occup Environ Med. 1997;54:254-63. |
| 11. | Stayner LT, Dankovic DA, Lemen RA. Occupational exposure to chrysotile asbestos and cancer risk: a review of the amphibole hypothesis. Am J Public Health. 1996;86(2):179-86. |
| 12. | Frumkin H, Berlin J. Asbestos exposure and gastrointestinal malignancy review and meta-analysis. Am J Ind Med. 1988;14:79-95. |
| 13. | Cantor KP. Drinking water and cancer. Cancer Causes Control. 1997;8:292–308. |
| 14. | Morgan RW, Foliart DE, Wong O. Asbestos and gastrointestinal cancer. A review of the literature. West J Med. 1985;143:60-5. |
| 15. | Pott F, Roller M, Ziem U, Reiffer FJ, Bellmann B, Rosenbruch M, et al. Carcinogenicity studies on natural and man-made fibres with the intraperitoneal test in rats. IARC Sci Publ. 1989;(90):173-9. |
| 16. | Berman DW, Crump KS. A meta-analysis of asbestos-related cancer risk that addresses fibre size and mineral type. Crit Rev Toxicol. 2008;38: Suppl 149-73. |
| 17. | Roggli VL. Human disease consequences of fiber exposures: a review of human lung pathology and fiber burden data. Environ Health Perspect. 1990;88:295-303. |
| 18. | Upton AC, Barret JC, Becklake MR, Burdett G, Chatfield E, Davis JMG, et al. Asbestos in public and commercial buildings: A literature review and synthesis of current knowledge [Website]. Cambridge, MA: Health Effects Institute. 1991, [cited Jun 18 2020]. Available in: https://www.healtheffects.org/system/files/SR_1991_Ch1-5_0.pdf https://www.healtheffects.org/system/files/SR_1991_Ch6-Appendix2_0.pdf https://www.healtheffects.org/system/files/SR_1991_Supplement_0.pdf |
| 19. | Camus M, Siemiatycki J, Meek B. Nonoccupational exposure to chrysotile asbestos and the risk of lung cancer. N Engl J Med. 1998;338(22):1565-71. |
| 20. | Hughes JM. Human evidence: lung cancer mortality risk from chrysotile exposure. Ann Occup Hyg. 1994;38(4):555-60, 415-6. |
| 21. | Landrigan PJ. Asbestos--still a carcinogen. N Engl J Med. 1998;338(22):1618-9. |
| 22. | Lanphear BP, Buncher CR. Latent period for malignant mesothelioma of occupational origin. J Occup Med. 1992;34(7):718-21. |
| 23. | Goodman M, Morgan RW, Ray R, Malloy CD, Zhao K. Cancer in asbestos-exposed occupational cohorts: a meta-analysis. Cancer Causes Control. 1999;10(5):453-65. |
| 24. | Gamble JF. Asbestos and colon cancer: a weight-of-the-evidence review. Environ Health Perspect. 1994;102(12):1038-50 |
| 25. | Nicholson WJ, Perkel G, Selikoff IJ. Occupational exposure to asbestos: population at risk and projected mortality--1980-2030. Am J Ind Med. 1982;3(3):259-311. |
| 26. | Peto J, Seidman H, Selikoff IJ. Mesothelioma mortality in asbestos workers: implications for models of carcinogenesis and risk assessment. Br J Cancer. 1982;45(1):124-35. |
| 27. | Peto J, Doll R, Hermon C, Binns W, Clayton R, Goffe T. Relationship of mortality to measures of environmental asbestos pollution in an asbestos textile factory. Ann Occup Hyg. 1985;29(3):305-55. |
| 28. | Edelman DA. Exposure to asbestos and the risk of gastrointestinal cancer: a reassessment. Br J Ind Med. 1988;45(2):75-82. |
| 29. | Edelman DA. Asbestos exposure, pleural plaques and the risk of lung cancer. Int Arch Occup Environ Health. 1988;60(6):389-93. |
| 30. | Edelman DA. Laryngeal cancer and occupational exposure to asbestos. Int Arch Occup Environ Health. 1989;61(4):223-7. |
| 31. | Weiss W. The lack of causality between asbestos and colorectal cancer. J Occup Environ Med. 1995;37(12):1364-73. |
| 32. | Kraus T, Drexler H, Weber A, Raithel HJ. The association of occupational asbestos dust exposure and laryngeal carcinoma. Isr J Med Sci. 1995;31(9):540-8. |
| 33. | Browne K, Gee JB. Asbestos exposure and laryngeal cancer. Ann Occup Hyg. 2000;44(4):239-50. |
| 34. | Kanarek MS. Epidemiological studies on ingested mineral fibres: gastric and other cancers. IARC Sci Publ. 1989;(90):428-37. |
| 35. | Stayner L, Smith R, Bailer J, Gilbert S, Steenland K, Dement J, et al. Exposure-response analysis of risk of respiratory disease associated with occupational exposure to chrysotile asbestos. Occup Environ Med. 1997;54(9):646-52. |
| 36. | Stayner L, Welch LS, Lemen R. The worldwide pandemic of asbestos-related diseases. Annu Rev Public Health. 2013;34:205-16. |
| 37. | Hein MJ, Stayner LT, Lehman E, Dement JM. Follow-up study of chrysotile textile workers: cohort mortality and exposure-response. Occup Environ Med. 2007 Sep;64(9):616-25. |
