# Supplementary material for: Fiber burden and asbestos-related diseases: an umbrella review
Source: Gac Sanit. Author manuscript; Available in PMC 2022 Mar 27. (PMC8882348; doi:10.1016/j.gaceta.2021.04.001)
Supplement: Supplemental Table 3 [file NIHMS1776636-supplement-Supplemental_Table_3.doc]

| Supplementary Table 3. References retrieved for each semantic search engine (syntax) in PubMed/Medline. | | | |  |  |
| --- | --- | --- | --- | --- | --- |
| **Syntax for lung cancer** | | | |  |  |
| Asbestos exposure AND lung cancer AND fiber concentration (n=16) | 1. | | Cox LA Jr. Dose-response modeling of NLRP3 inflammasome-mediated diseases: asbestos, lung cancer, and malignant mesothelioma as examples. Crit Rev Toxicol. 2019;49:614-635. |  |  |
|  | 2. | | Yano E. Adverse health effects of asbestos: solving mysteries regarding asbestos carcinogenicity based on follow-up survey of a Chinese factory. Environ Health Prev Med. 2018;23(1):35. |  |  |
|  | 3. | | Roggli VL. The So-called Short-Fiber Controversy: Literature Review and Critical Analysis. Arch Pathol Lab Med. 2015;139(8):1052-7. |  |  |
|  | 4. | | Moolgavkar SH, Anderson EL, Chang ET, Lau EC, Turnham P, Hoel DG. A review and critique of U.S. EPA's risk assessments for asbestos. Crit Rev Toxicol. 2014;44(6):499-522. |  |  |
|  | 5. | | Berman DW, Crump KS. A meta-analysis of asbestos-related cancer risk that addresses fiber size and mineral type. Crit Rev Toxicol. 2008;38 Suppl 1:49-73. |  |  |
|  | 6. | | Britton M. The epidemiology of mesothelioma. Semin Oncol. 2002;29(1):18-25. |  |  |
|  | 7. | | Maxim LD, McConnell EE. Interspecies comparisons of the toxicity of asbestos and synthetic vitreous fibers: a weight-of-the-evidence approach. Regul Toxicol Pharmacol. 2001;33(3):319-42. |  |  |
|  | 8. | | Roggli VL. Human disease consequences of fiber exposures: a review of human lung pathology and fiberburden data. Environ Health Perspect. 1990;88:295-303. |  |  |
|  | 9. | | Merchant JA. Human epidemiology: a review of fiber type and characteristics in the development of malignant and nonmalignant disease. Environ Health Perspect. 1990;88:287-93. |  |  |
|  | 10. | | Talcott JA, Antman KH. Asbestos-related malignancy. Curr Probl Cancer. 1988;12(3):135-78. |  |  |
|  | 11. | | Hughes JM, Weill H. Asbestos exposure--quantitative assessment of risk. Am Rev Respir Dis. 1986;133(1):5-13. |  |  |
|  | 12. | | Lee KP. Lung response to particulates with emphasis on asbestos and other fibrous dusts. Crit Rev Toxicol. 1985;14(1):33-86. |  |  |
|  | 13. | | Kannerstein M, Churg J. Mesothelioma in man and experimental animals. Environ Health Perspect. 1980;34:31-6. |  |  |
|  | 14. | | Utell MJ, Maxim LD. Refractory ceramic fibers: Fiber characteristics, potential health effects and clinical observations. Toxicol Appl Pharmacol. 2018;361:113-117. |  |  |
|  | 15. | | Asgharian B, Owen TP, Kuempel ED, Jarabek AM. Dosimetry of inhaled elongate mineral particles in the respiratory tract: The impact of shape factor. Toxicol Appl Pharmacol. 2018 Dec 15;361:27-35. |  |  |
|  | 16. | | Oberdörster G. Determinants of the pathogenicity of man-made vitreous fibers (MMVF). Int Arch Occup Environ Health. 2000 Jun;73 Suppl:S60-8. |  |  |
| Asbestos exposure AND lung cancer AND dose-exposure (n=2) | 1. | | Falaschi F, Romei C, Fiorini S, Lucchi M. Imaging of malignant pleural mesothelioma: it is possible a screening or early diagnosis program?-a systematic review about the use of screening programs in a population of asbestos exposed workers. J Thorac Dis. 2018;10(Suppl 2):S262-S268. |  |  |
|  | 2. | | Case BW, Abraham JL, Meeker G, Pooley FD, Pinkerton KE. Applying definitions of "asbestos" to environmental and "low-dose" exposure levels and health effects, particularly malignant mesothelioma. J Toxicol Environ Health B Crit Rev. 2011;14(1-4):3-39. |  |  |
| Asbestos exposure AND lung cancer AND exposure-response (n=13) | 1. | | Marsh GM, Riordan AS, Keeton KA, Benson SM. Non-occupational exposure to asbestos and risk of pleural mesothelioma: review and meta-analysis. Occup Environ Med. 2017;74(11):838-846. |  |  |
|  | 2. | | Moolgavkar SH, Anderson EL, Chang ET, Lau EC, Turnham P, Hoel DG. A review and critique of U.S. EPA's risk assessments for asbestos. Crit Rev Toxicol. 2014;44(6):499-522. |  |  |
|  | 3. | | van der Bij S, Koffijberg H, Lenters V, Portengen L, Moons KG, Heederik D, et al. Lung cancer risk at low cumulative asbestos exposure: meta-regression of the exposure-response relationship. Cancer Causes Control. 2013;24(1):1-12. |  |  |
|  | 4. | | Finley BL, Pierce JS, Phelka AD, Adams RE, Paustenbach DJ, Thuett KA, et al. Evaluation of tremolite asbestos exposures associated with the use of commercial products. Crit Rev Toxicol. 2012;42(2):119-46. Erratum in: Crit Rev Toxicol. 2018;48(5):416. |  |  |
|  | 5. | | Antao VC, Larson TC, Horton DK. Libby vermiculite exposure and risk of developing asbestos-related lung and pleural diseases. Curr Opin Pulm Med. 2012;18(2):161-7. |  |  |
|  | 6. | | Lenters V, Vermeulen R, Dogger S, Stayner L, Portengen L, Burdorf A, et al. A meta-analysis of asbestos and lung cancer: is better quality exposure assessment associated with steeper slopes of the exposure-response relationships?. Environ Health Perspect. 2011;119(11):1547-55. |  |  |
|  | 7. | | Case BW, Abraham JL, Meeker G, Pooley FD, Pinkerton KE. Applying definitions of "asbestos" to environmental and "low-dose" exposure levels and health effects, particularly malignant mesothelioma. J Toxicol Environ Health B Crit Rev. 2011;14(1-4):3-39. |  |  |
|  | 8. | | Berman DW. Comparing milled fiber, Quebec ore, and textile factory dust: has another piece of the asbestos puzzle fallen into place? Crit Rev Toxicol. 2010;40(2):151-88. |  |  |
|  | 9. | | Gamble JF, Gibbs GW. An evaluation of the risks of lung cancer and mesothelioma from exposure to amphibole cleavage fragments. Regul Toxicol Pharmacol. 2008 Oct;52(1 Suppl):S154-86. |  |  |
|  | 10. | | Pierce JS, McKinley MA, Paustenbach DJ, Finley BL. An evaluation of reported no-effect chrysotile asbestos exposures for lung cancer and mesothelioma. Crit Rev Toxicol. 2008;38(3):191-214. |  |  |
|  | 11. | | Gamble J. Risk of gastrointestinal cancers from inhalation and ingestion of asbestos. Regul Toxicol Pharmacol. 2008;52(1 Suppl):S124-53. |  |  |
|  | 12. | | Berry G, Gibbs GW. An overview of the risk of lung cancer in relation to exposure to asbestos and of taconite miners. Regul Toxicol Pharmacol. 2008;52(1 Suppl):S218-22. |  |  |
|  | 13. | | Gamble JF. Asbestos and colon cancer: a weight-of-the-evidence review. Environ Health Perspect. 1994;102(12):1038-50. |  |  |
| Asbestos exposure AND lung cancer AND dose-response (n=24) | 1. | | Cox LA Jr. Dose-response modeling of NLRP3 inflammasome-mediated diseases: asbestos, lung cancer, and malignant mesothelioma as examples. Crit Rev Toxicol. 2019;49:614-635. |  |  |
|  | 2. | | Benvenuto M, Mattera R, Taffera G, Giganti MG, Lido P, Masuelli L, et al. The Potential Protective Effects of Polyphenols in Asbestos-Mediated Inflammation and Carcinogenesis of Mesothelium. Nutrients. 2016;8(5). |  |  |
|  | 3. | | Schwartz AG, Cote ML. Epidemiology of Lung Cancer. Adv Exp Med Biol. 2016;893:21-41. |  |  |
|  | 4. | | Delva F, Andujar P, Lacourt A, Brochard P, Pairon JC. Occupational risk factors for lung cancer. Rev Mal Respir. 2016;33(6):444-59. |  |  |
|  | 5. | | Moolgavkar SH, Anderson EL, Chang ET, Lau EC, Turnham P, Hoel DG. A review and critique of U.S. EPA's risk assessments for asbestos. Crit Rev Toxicol. 2014;44(6):499-522. |  |  |
|  | 6. | | Bernstein D, Dunnigan J, Hesterberg T, Brown R, Velasco JA, Barrera R, et al. Health risk of chrysotile revisited. Crit Rev Toxicol. 2013;43(2):154-83. |  |  |
|  | 7. | | Mossman BT, Lippmann M, Hesterberg TW, Kelsey KT, Barchowsky A, Bonner JC. Pulmonary endpoints (lung carcinomas and asbestosis) following inhalation exposure to asbestos. J Toxicol Environ Health B Crit Rev. 2011;14(1-4):76-121. |  |  |
|  | 8. | | Case BW, Abraham JL, Meeker G, Pooley FD, Pinkerton KE. Applying definitions of "asbestos" to environmental and "low-dose" exposure levels and health effects, particularly malignant mesothelioma. J Toxicol Environ Health B Crit Rev. 2011;14(1-4):3-39. |  |  |
|  | 9. | | Merler E. Mesothelioma incidence decreases parallel to asbestos exposure decrement or interruption: a confirmation of a dose-response relationship, with implications in public health. Epidemiol Prev. 2007;31(4 Suppl 1):46-52. |  |  |
|  | 10. | | Berry G, Gibbs GW. An overview of the risk of lung cancer in relation to exposure to asbestos and of taconite miners. Regul Toxicol Pharmacol. 2008;52(1 Suppl):S218-22. |  |  |
|  | 11. | | Bernstein DM, Hoskins JA. The health effects of chrysotile: current perspective based upon recent data. Regul Toxicol Pharmacol. 2006;45(3):252-64. |  |  |
|  | 12. | | Russell RM. The enigma of beta-carotene in carcinogenesis: what can be learned from animal studies. J Nutr. 2004;134(1):262S-268S. |  |  |
|  | 13. | | Goodman M, Morgan RW, Ray R, Malloy CD, Zhao K. Cancer in asbestos-exposed occupational cohorts: a meta-analysis. Cancer Causes Control. 1999;10(5):453-65. |  |  |
|  | 14. | | Oberdörster G. Pulmonary carcinogenicity of inhaled particles and the maximum tolerated dose. Environ Health Perspect. 1997;105 Suppl 5:1347-55. |  |  |
|  | 15. | | Hillerdal G, Henderson DW. Asbestos, asbestosis, pleural plaques and lung cancer. Scand J Work Environ Health. 1997;23(2):93-103. |  |  |
|  | 16. | | Lash TL, Crouch EA, Green LC. A meta-analysis of the relation between cumulative exposure to asbestos and relative risk of lung cancer. Occup Environ Med. 1997;54(4):254-63. |  |  |
|  | 17. | | Whitesell PL, Drage CW. Occupational lung cancer. Mayo Clin Proc. 1993;68(2):183-8. |  |  |
|  | 18. | | Nicholson WJ. Comparative dose-response relationships of asbestos fiber types: magnitudes and uncertainties. Ann N Y Acad Sci. 1991;643:74-84. |  |  |
|  | 19. | | Smith AH, Handley MA, Wood R. Epidemiological evidence indicates asbestos causes laryngeal cancer. J Occup Med. 1990 Jun;32(6):499-507. |  |  |
|  | 20. | | Lippmann M. Asbestos exposure indices. Environ Res. 1988;46(1):86-106. |  |  |
|  | 21. | | Churg A. Chrysotile, tremolite, and malignant mesothelioma in man. Chest. 1988;93(3):621-8. |  |  |
|  | 22. | | Huncharek M. The biomedical and epidemiological characteristics of asbestos-related diseases: a review. Yale J Biol Med. 1986;59(4):435-51. |  |  |
|  | 23. | | Hughes JM, Weill H. Asbestos exposure--quantitative assessment of risk. Am Rev Respir Dis. 1986;133(1):5-13. |  |  |
|  | 24. | | Rom WN, Lockey JE. Diffuse malignant mesothelioma: a review. West J Med. 1982;137(6):548-54. |  |  |
| **Syntax for lung mesothelioma** | | | |  |  |
| Asbestos exposure AND mesothelioma AND fiber concentration (n=16) | 1. | | Cheng TJ, More SL, Maddaloni MA, Fung ES. Evaluation of potential gastrointestinal carcinogenicity associated with the ingestion of asbestos. Rev Environ Health. 2020 Sep 23;36:15-26. |  |  |
|  | 2. | | Cox LA Jr. Dose-response modeling of NLRP3 inflammasome-mediated diseases: asbestos, lung cancer, and malignant mesothelioma as examples. Crit Rev Toxicol. 2019;49:614-635. |  |  |
|  | 3. | | Roggli VL. The So-called Short-Fiber Controversy: Literature Review and Critical Analysis. Arch Pathol Lab Med. 2015;139(8):1052-7. |  |  |
|  | 4. | | Banks DE. Clinical aspects of asbestos-related diseases--what are the unresolved topics? J Occup Environ Med. 2014;56 Suppl 10:S8-S12. |  |  |
|  | 5. | | Moolgavkar SH, Anderson EL, Chang ET, Lau EC, Turnham P, Hoel DG. A review and critique of U.S. EPA's risk assessments for asbestos. Crit Rev Toxicol. 2014;44(6):499-522. |  |  |
|  | 6. | | Berman DW, Crump KS. A meta-analysis of asbestos-related cancer risk that addresses fiber size and mineral type. Crit Rev Toxicol. 2008;38 Suppl 1:49-73. |  |  |
|  | 7. | | Britton M. The epidemiology of mesothelioma. Semin Oncol. 2002;29(1):18-25. |  |  |
|  | 8. | | Roggli VL. Human disease consequences of fiber exposures: a review of human lung pathology and fiberburden data. Environ Health Perspect. 1990;88:295-303. |  |  |
|  | 9. | | Merchant JA. Human epidemiology: a review of fiber type and characteristics in the development of malignant and nonmalignant disease. Environ Health Perspect. 1990;88:287-93. |  |  |
|  | 10. | | Talcott JA, Antman KH. Asbestos-related malignancy. Curr Probl Cancer. 1988;12(3):135-78. |  |  |
|  | 11. | | Hughes JM, Weill H. Asbestos exposure--quantitative assessment of risk. Am Rev Respir Dis. 1986;133(1):5-13. |  |  |
|  | 12. | | Lee KP. Lung response to particulates with emphasis on asbestos and other fibrous dusts. Crit Rev Toxicol. 1985;14(1):33-86. |  |  |
|  | 13. | | Kannerstein M, Churg J. Mesothelioma in man and experimental animals. Environ Health Perspect. 1980;34:31-6. |  |  |
|  | 14. | | Utell MJ, Maxim LD. Refractory ceramic fibers: Fiber characteristics, potential health effects and clinical observations. Toxicol Appl Pharmacol. 2018;361:113-117. |  |  |
|  | 15. | | Asgharian B, Owen TP, Kuempel ED, Jarabek AM. Dosimetry of inhaled elongate mineral particles in the respiratory tract: The impact of shape factor. Toxicol Appl Pharmacol. 2018;361:27-35. |  |  |
|  | 16. | | Yarborough CM. Chrysotile as a cause of mesothelioma: an assessment based on epidemiology. Crit Rev Toxicol. 2006;36(2):165-87. |  |  |
| Asbestos exposure AND mesothelioma AND dose-exposure (n=4) | 1. | | Falaschi F, Romei C, Fiorini S, Lucchi M. Imaging of malignant pleural mesothelioma: it is possible a screening or early diagnosis program? A systematic review about the use of screening programs in a population of asbestos exposed workers. J Thorac Dis. 2018;10(Suppl 2):S262-S268. |  |  |
|  | 2. | | Musk ABW, de Klerk N, Brims FJ. Mesothelioma in Australia: a review. Med J Aust. 2017;207(10):449-452. |  |  |
|  | 3. | | Case BW, Abraham JL, Meeker G, Pooley FD, Pinkerton KE. Applying definitions of "asbestos" to environmental and "low-dose" exposure levels and health effects, particularly malignant mesothelioma. J Toxicol Environ Health B Crit Rev. 2011;14(1-4):3-39. |  |  |
|  | 4. | | Hillerdal G. Mesothelioma: cases associated with non-occupational and low dose exposures. Occup Environ Med. 1999;56(8):505-13. |  |  |
| Asbestos exposure AND mesothelioma AND exposure-response (n=11) | 1. | | Marsh GM, Riordan AS, Keeton KA, Benson SM. Non-occupational exposure to asbestos and risk of pleural mesothelioma: review and meta-analysis. Occup Environ Med. 2017;74(11):838-846. |  |  |
|  | 2. | | Moolgavkar SH, Anderson EL, Chang ET, Lau EC, Turnham P, Hoel DG. A review and critique of U.S. EPA's risk assessments for asbestos. Crit Rev Toxicol. 2014;44(6):499-522. |  |  |
|  | 3. | | van der Bij S, Koffijberg H, Lenters V, Portengen L, Moons KG, et al. Lung cancer risk at low cumulative asbestos exposure: meta-regression of the exposure-response relationship. Cancer Causes Control. 2013;24(1):1-12. |  |  |
|  | 4. | | Finley BL, Pierce JS, Phelka AD, Adams RE, Paustenbach DJ, et al. Evaluation of tremolite asbestos exposures associated with the use of commercial products. Crit Rev Toxicol. 2012;42(2):119-46. Erratum in: Crit Rev Toxicol. 2018;48(5):416. |  |  |
|  | 5. | | Reid A, de Klerk N, Musk AW. Does exposure to asbestos cause ovarian cancer? A systematic literature review and meta-analysis. Cancer Epidemiol Biomarkers Prev. 2011;20(7):1287-95. |  |  |
|  | 6. | | Case BW, Abraham JL, Meeker G, Pooley FD, Pinkerton KE. Applying definitions of "asbestos" to environmental and "low-dose" exposure levels and health effects, particularly malignant mesothelioma. J Toxicol Environ Health B Crit Rev. 2011;14(1-4):3-39. |  |  |
|  | 7. | | Berman DW. Comparing milled fiber, Quebec ore, and textile factory dust: has another piece of the asbestos puzzle fallen into place? Crit Rev Toxicol. 2010;40(2):151-88. |  |  |
|  | 8. | | Gamble JF, Gibbs GW. An evaluation of the risks of lung cancer and mesothelioma from exposure to amphibole cleavage fragments. Regul Toxicol Pharmacol. 2008;52(1 Suppl):S154-86. |  |  |
|  | 9. | | Pierce JS, McKinley MA, Paustenbach DJ, Finley BL. An evaluation of reported no-effect chrysotile asbestos exposures for lung cancer and mesothelioma. Crit Rev Toxicol. 2008;38(3):191-214. |  |  |
|  | 10. | | Gamble J. Risk of gastrointestinal cancers from inhalation and ingestion of asbestos. Regul Toxicol Pharmacol. 2008;52(1 Suppl):S124-53. |  |  |
|  | 11. | | Berry G, Gibbs GW. An overview of the risk of lung cancer in relation to exposure to asbestos and of taconite miners. Regul Toxicol Pharmacol. 2008;52(1 Suppl):S218-22. |  |  |
| Asbestos exposure AND mesothelioma AND dose-response (n=21) | 1. | | Korchevskiy A. Using benchmark dose modeling for the quantitative risk assessment: Carbon nanotubes, asbestos, glyphosate. J Appl Toxicol. 2021;41:148-160. |  |  |
|  | 2. | | Cox LAT Jr. Biological mechanisms of non-linear dose-response for respirable mineral fibers. Toxicol Appl Pharmacol. 2018;361:137-144. |  |  |
|  | 3. | | Cox LA Jr. Dose-response modeling of NLRP3 inflammasome-mediated diseases: asbestos, lung cancer, and malignant mesothelioma as examples. Crit Rev Toxicol. 2019;49:614-635. |  |  |
|  | 4. | | Benvenuto M, Mattera R, Taffera G, Giganti MG, Lido P, et al. The Potential Protective Effects of Polyphenols in Asbestos-Mediated Inflammation and Carcinogenesis of Mesothelium. Nutrients. 2016;8:275. |  |  |
|  | 5. | | Moolgavkar SH, Anderson EL, Chang ET, Lau EC, Turnham P, et al. A review and critique of U.S. EPA's risk assessments for asbestos. 2014;44(6):499-522. |  |  |
|  | 6. | | Bernstein D, Dunnigan J, Hesterberg T, Brown R, Velasco JA, et al. Health risk of chrysotile revisited. Crit Rev Toxicol. 2013 Feb;43(2):154-83. |  |  |
|  | 7. | | Mossman BT, Lippmann M, Hesterberg TW, Kelsey KT, Barchowsky A, et al. Pulmonary endpoints (lung carcinomas and asbestosis) following inhalation exposure to asbestos. J Toxicol Environ Health B Crit Rev. 2011;14(1-4):76-121. |  |  |
|  | 8. | | Case BW, Abraham JL, Meeker G, Pooley FD, Pinkerton KE. Applying definitions of "asbestos" to environmental and "low-dose" exposure levels and health effects, particularly malignant mesothelioma. J Toxicol Environ Health B Crit Rev. 2011;14(1-4):3-39. |  |  |
|  | 9. | | Merler E. Mesothelioma incidence decreases parallel to asbestos exposure decrement or interruption: a confirmation of a dose-response relationship, with implications in public health. Epidemiol Prev. 2007;31(4 Suppl 1):46-52. |  |  |
|  | 10. | | Gibbs GW, Berry G. Mesothelioma and asbestos. Regul Toxicol Pharmacol. 2008;52(1 Suppl):S223-31. |  |  |
|  | 11. | | Berry G, Gibbs GW. An overview of the risk of lung cancer in relation to exposure to asbestos and of taconite miners. Regul Toxicol Pharmacol. 2008;52(1 Suppl):S218-22. |  |  |
|  | 12. | | Tomatis L, Cantoni S, Carnevale F, Merler E, Mollo F, et al. The role of asbestos fiber dimensions in the prevention of mesothelioma. Int J Occup Environ Health. 2007;13(1):64-9. |  |  |
|  | 13. | | Tomatis L, Cantoni S, Carnevale F, Merler E, Mollo F, et al. The role of asbestos fibre dimensions in the pathogenesis and prevention of mesothelioma. Epidemiol Prev. 2006;30(4-5):289-94. |  |  |
|  | 14. | | Bernstein DM, Hoskins JA. The health effects of chrysotile: current perspective based upon recent data. Regul Toxicol Pharmacol. 2006;45(3):252-64. |  |  |
|  | 15. | | Goodman M, Morgan RW, Ray R, Malloy CD, Zhao K. Cancer in asbestos-exposed occupational cohorts: a meta-analysis. Cancer Causes Control. 1999;10(5):453-65. |  |  |
|  | 16. | | Smith AH, Handley MA, Wood R. Epidemiological evidence indicates asbestos causes laryngeal cancer. J Occup Med. 1990;32(6):499-507. |  |  |
|  | 17. | | Lippmann M. Asbestos exposure indices. Environ Res. 1988;46(1):86-106. |  |  |
|  | 18. | | Churg A. Chrysotile, tremolite, and malignant mesothelioma in man. Chest. 1988;93(3):621-8. |  |  |
|  | 19. | | Huncharek M. The biomedical and epidemiological characteristics of asbestos-related diseases: a review. Yale J Biol Med. 1986;59(4):435-51. |  |  |
|  | 20. | | Hughes JM, Weill H. Asbestos exposure--quantitative assessment of risk. Am Rev Respir Dis. 1986;133(1):5-13. |  |  |
|  | 21. | | Rom WN, Lockey JE. Diffuse malignant mesothelioma: a review. West J Med. 1982;137(6):548-54. |  |  |
| **Syntax for asbestosis** | | | |  |  |
| Asbestos exposure AND asbestosis AND fiber concentration (n=11) | 1. | | Roggli VL. The So-called Short-Fiber Controversy: Literature Review and Critical Analysis. Arch Pathol Lab Med. 2015;139(8):1052-7. |  |  |
|  | 2. | | Banks DE. Clinical aspects of asbestos-related diseases--what are the unresolved topics? J Occup Environ Med. 2014;56 Suppl 10:S8-S12. |  |  |
|  | 3. | | Hollins DM, Paustenbach DJ, Clark K, Mangold CA. A visual historical review of exposure to asbestos at puget sound naval shipyard (1962-1972). J Toxicol Environ Health B Crit Rev. 2009;12(2):124-56. Erratum in: J Toxicol Environ Health B Crit Rev. 2009;12(3):224. |  |  |
|  | 4. | | Williams PR, Phelka AD, Paustenbach DJ. A review of historical exposures to asbestos among skilled craftsmen (1940-2006). J Toxicol Environ Health B Crit Rev. 2007;10(5):319-77. |  |  |
|  | 5. | | Bhattacharya K, Dopp E, Kakkar P, Jaffery FN, Schiffmann D, et al. Biomarkers in risk assessment of asbestos exposure. Mutat Res. 2005;579(1-2):6-21. |  |  |
|  | 6. | | Maxim LD, McConnell EE. Interspecies comparisons of the toxicity of asbestos and synthetic vitreous fibers: a weight-of-the-evidence approach. Regul Toxicol Pharmacol. 2001;33(3):319-42. |  |  |
|  | 7. | | Roggli VL. Human disease consequences of fiber exposures: a review of human lung pathology and fiber burden data. Environ Health Perspect. 1990;88:295-303. |  |  |
|  | 8. | | Merchant JA. Human epidemiology: a review of fiber type and characteristics in the development of malignant and nonmalignant disease. Environ Health Perspect. 1990;88:287-93. |  |  |
|  | 9. | | Hughes JM, Weill H. Asbestos exposure--quantitative assessment of risk. Am Rev Respir Dis. 1986;133(1):5-13. |  |  |
|  | 10. | | Lee KP. Lung response to particulates with emphasis on asbestos and other fibrous dusts. Crit Rev Toxicol. 1985;14(1):33-86. |  |  |
|  | 11. | | Asgharian B, Owen TP, Kuempel ED, Jarabek AM. Dosimetry of inhaled elongate mineral particles in the respiratory tract: The impact of shape factor. Toxicol Appl Pharmacol. 2018 Dec 15;361:27-35. |  |  |
| Asbestos exposure AND asbestosis AND dose-exposure (n=1) | 1. | | Musk ABW, de Klerk N, Brims FJ. Mesothelioma in Australia: a review. Med J Aust. 2017;207(10):449-452. |  |  |
| Asbestos exposure AND asbestosis AND exposure-response (n=5) | 1. | | Barlow CA, Sahmel J, Paustenbach DJ, Henshaw JL. History of knowledge and evolution of occupational health and regulatory aspects of asbestos exposure science: 1900-1975. Crit Rev Toxicol. 2017;47(4):286-316. |  |  |
|  | 2. | | van der Bij S, Koffijberg H, Lenters V, Portengen L, Moons KG, et al. Lung cancer risk at low cumulative asbestos exposure: meta-regression of the exposure-response relationship. Cancer Causes Control. 2013;24(1):1-12. |  |  |
|  | 3. | | Antao VC, Larson TC, Horton DK. Libby vermiculite exposure and risk of developing asbestos-related lung and pleural diseases. Curr Opin Pulm Med. 2012;18(2):161-7. |  |  |
|  | 4. | | Gamble JF, Gibbs GW. An evaluation of the risks of lung cancer and mesothelioma from exposure to amphibole cleavage fragments. Regul Toxicol Pharmacol. 2008;52(1 Suppl):S154-86. |  |  |
|  | 5. | | Gamble J. Risk of gastrointestinal cancers from inhalation and ingestion of asbestos. Regul Toxicol Pharmacol. 2008;52(1 Suppl):S124-53. |  |  |
| Asbestos exposure AND asbestosis AND dose-response (n=11) | 1. | | Bernstein D, Dunnigan J, Hesterberg T, Brown R, Velasco JA, et al. Health risk of chrysotile revisited. Crit Rev Toxicol. 2013;43(2):154-83. |  |  |
|  | 2. | | Mossman BT, Lippmann M, Hesterberg TW, Kelsey KT, Barchowsky A, et al. Pulmonary endpoints (lung carcinomas and asbestosis) following inhalation exposure to asbestos. J Toxicol Environ Health B Crit Rev. 2011;14(1-4):76-121 |  |  |
|  | 3. | | Baur X, Manuwald U, Wilken D. Does long-term asbestos exposure cause an obstructive ventilation pattern? Pneumologie. 2010;64(12):736-44. |  |  |
|  | 4. | | Gibbs GW, Berry G. Mesothelioma and asbestos. Regul Toxicol Pharmacol. 2008;52(1 Suppl):S223-31. |  |  |
|  | 5. | | Hillerdal G, Henderson DW. Asbestos, asbestosis, pleural plaques and lung cancer. Scand J Work Environ Health. 1997;23(2):93-103. |  |  |
|  | 6. | | Becklake MR, Case BW. Fiber burden and asbestos-related lung disease: determinants of dose-response relationships. Am J Respir Crit Care Med. 1994;150(6 Pt 1):1488-92. |  |  |
|  | 7. | | Lippmann M. Asbestos exposure indices. Environ Res. 1988;46(1):86-106. |  |  |
|  | 8. | | Churg A. Chrysotile, tremolite, and malignant mesothelioma in man. Chest. 1988;93(3):621-8. |  |  |
|  | 9. | | Huncharek M. The biomedical and epidemiological characteristics of asbestos-related diseases: a review. Yale J Biol Med. 1986;59(4):435-51. |  |  |
|  | 10. | | Hughes JM, Weill H. Asbestos exposure--quantitative assessment of risk. Am Rev Respir Dis. 1986;133(1):5-13. |  |  |
|  | 11. | | Rom WN, Lockey JE. Diffuse malignant mesothelioma: a review. West J Med. 1982;137(6):548-54. |  |  |
| **Syntax for diffuse interstitial pulmonary fibrosis** | | | |  |  |
| Asbestos exposure AND diffuse interstitial pulmonary fibrosis AND fiber concentration (n=5) | 1. | | Roggli VL. The So-called Short-Fiber Controversy: Literature Review and Critical Analysis. Arch Pathol Lab Med. 2015;139(8):1052-7. |  |  |
|  | 2. | | Roggli VL. Human disease consequences of fiber exposures: a review of human lung pathology and fiberburden data. Environ Health Perspect. 1990;88:295-303. |  |  |
|  | 3. | | Merchant JA. Human epidemiology: a review of fiber type and characteristics in the development of malignant and nonmalignant disease. Environ Health Perspect. 1990;88:287-93. |  |  |
|  | 4. | | Talcott JA, Antman KH. Asbestos-related malignancy. Curr Probl Cancer. 1988;12(3):135-78. |  |  |
|  | 5. | | Lee KP. Lung response to particulates with emphasis on asbestos and other fibrous dusts. Crit Rev Toxicol. 1985;14(1):33-86. |  |  |
| Asbestos exposure AND diffuse interstitial pulmonary fibrosis AND dose-exposure (n=0) | | | |  |  |
| Asbestos exposure AND diffuse interstitial pulmonary fibrosis AND exposure-response (n=0) | | | |  |  |
| Asbestos exposure AND diffuse interstitial pulmonary fibrosis AND dose-response  (n=2) | 1. | | Mossman BT, Lippmann M, Hesterberg TW, Kelsey KT, Barchowsky A, Bonner JC. Pulmonary endpoints (lung carcinomas and asbestosis) following inhalation exposure to asbestos. J Toxicol Environ Health B Crit Rev. 2011;14(1-4):76-121. |  |  |
|  | 2. | | Hillerdal G, Henderson DW. Asbestos, asbestosis, pleural plaques and lung cancer. Scand J Work Environ Health. 1997;23(2):93-103. |  |  |
| **Syntax for pleural plaques** | | | |  |  |
| Asbestos exposure AND pleural plaques AND fiber concentration (n=10) | 1. | | Asgharian B, Owen TP, Kuempel ED, Jarabek AM. Dosimetry of inhaled elongate mineral particles in the respiratory tract: The impact of shape factor. Toxicol Appl Pharmacol. 2018;361:27-35. |  |  |
|  | 2. | | Utell MJ, Maxim LD. Refractory ceramic fibers: Fiber characteristics, potential health effects and clinical observations. Toxicol Appl Pharmacol. 2018;361:113-117. |  |  |
|  | 3. | | Greim H, Utell MJ, Maxim LD, Niebo R. Perspectives on refractory ceramic fiber (RCF) carcinogenicity: comparisons with other fibers. Inhal Toxicol. 2014;26:789-810 |  |  |
|  | 4. | | Moolgavkar SH, Anderson EL, Chang ET, Lau EC, Turnham P, et al. A review and critique of U.S. EPA's risk assessments for asbestos. Crit Rev Toxicol. 2014;44:499-522. |  |  |
|  | 5. | | Bhattacharya K, Dopp E, Kakkar P, Jaffery FN, Schiffmann D, et al. Biomarkers in risk assessment of asbestos exposure. Mutat Res. 2005;579(1-2):6-21. |  |  |
|  | 6. | | Chiappino G. Mesotelioma: il ruolo delle fibre ultrafini e conseguenti riflessi in campo preventivo e medico legale [Mesothelioma: the aetiological role of ultrathin fibres and repercussions on prevention and medical legal evaluation]. Med Lav. 2005;96:3-23. |  |  |
|  | 7. | | Peacock C, Copley SJ, Hansell DM. Asbestos-related benign pleural disease. Clin Radiol. 2000;55:422-32. |  |  |
|  | 8. | | Nishimura SL, Broaddus VC. Asbestos-induced pleural disease. Clin Chest Med. 1998;19:311-29. |  |  |
|  | 9. | | Case BW. Health effects of tremolite. Now and in the future. Ann N Y Acad Sci. 1991;643:491-504. |  |  |
|  | 10. | | Merchant JA. Human epidemiology: a review of fiber type and characteristics in the development of malignant and nonmalignant disease. Environ Health Perspect. 1990;88:287-93. |  |  |
| Asbestos exposure AND pleural plaques AND dose-exposure (n=0) | | | |  |  |
| Asbestos exposure AND pleural plaques AND exposure-response (n=2) | 1. | | Moolgavkar SH, Anderson EL, Chang ET, Lau EC, Turnham P, et al. A review and critique of U.S. EPA's risk assessments for asbestos. Crit Rev Toxicol. 2014;44(6):499-522. |  |  |
|  | 2. | | Antao VC, Larson TC, Horton DK. Libby vermiculite exposure and risk of developing asbestos-related lung and pleural diseases. Curr Opin Pulm Med. 2012;18(2):161-7. |  |  |
| Asbestos exposure AND pleural plaques AND dose-response (n=4) | 1. | | Moolgavkar SH, Anderson EL, Chang ET, Lau EC, Turnham P, et al. A review and critique of U.S. EPA's risk assessments for asbestos. Crit Rev Toxicol. 2014;44(6):499-522. |  |  |
|  | 2. | | Boffetta P. Health effects of asbestos exposure in humans: a quantitative assessment. Med Lav. 1998;89:471-80. |  |  |
|  | 3. | | Hillerdal G, Henderson DW. Asbestos, asbestosis, pleural plaques and lung cancer. Scand J Work Environ Health. 1997;23(2):93-103. |  |  |
|  | 4. | | Rom WN, Lockey JE. Diffuse malignant mesothelioma: a review. West J Med. 1982;137(6):548-54. |  |  |
| **Syntax for diffuse pleural fibrosis** | | | |  |  |
| Asbestos exposure AND diffuse pleural fibrosis AND fiber concentration (n=1) | 1. | | Merchant JA. Human epidemiology: a review of fiber type and characteristics in the development of malignant and nonmalignant disease. Environ Health Perspect. 1990;88:287-93. |  |  |
| Asbestos exposure AND diffuse pleural fibrosis AND dose-exposure (n=0) | | | |  |  |
| Asbestos exposure AND diffuse pleural fibrosis AND exposure-response (n=0) | | | |  |  |
| Asbestos exposure AND diffuse pleural fibrosis AND dose-response (n=0) | | | |  |  |
| **Syntax for laryngeal cancer** | | | |  |  |
| Asbestos exposure AND laryngeal cancer AND fiber concentration (n=0) | | | |  |  |
| Asbestos exposure AND laryngeal cancer AND dose-exposure (n=0) | | | |  |  |
| Asbestos exposure AND laryngeal cancer AND exposure-response (n=0) | | | |  |  |
| Asbestos exposure AND laryngeal cancer AND dose-response (n=2) | 1. | | Goodman M, Morgan RW, Ray R, Malloy CD, Zhao K. Cancer in asbestos-exposed occupational cohorts: a meta-analysis. Cancer Causes Control. 1999;10(5):453-65. |  |  |
|  | 2. | | Smith AH, Handley MA, Wood R. Epidemiological evidence indicates asbestos causes laryngeal cancer. J Occup Med. 1990;32(6):499-507. |  |  |
| **Syntax for gastrointestinal cancer** | | | |  |  |
| Asbestos exposure AND gastrointestinal cancer AND fiber concentration  (n=3) | | 1. | Cheng TJ, More SL, Maddaloni MA, Fung ES. Evaluation of potential gastrointestinal carcinogenicity associated with the ingestion of asbestos. Rev Environ Health. 2020;36:15-26. |  |  |
|  |  | 2. | Di Ciaula A. Asbestos ingestion and gastrointestinal cancer: a possible underestimated hazard. Expert Rev Gastroenterol Hepatol. 2017;11:419-425. |  |  |
|  |  | 3. | Richter ED. Asbestos exposure in Israel: findings, issues and needs. Isr J Med Sci. 1984;20:89-97. |  |  |
| Asbestos exposure AND gastrointestinal cancer AND dose-exposure (n=0) | | | |  |  |
| Asbestos exposure AND gastrointestinal cancer AND exposure-response (n=2) | | 1. | Gamble J. Risk of gastrointestinal cancers from inhalation and ingestion of asbestos. Regul Toxicol Pharmacol. 2008 Oct;52(1 Suppl):S124-53. |  |  |
|  |  | 2. | Gamble JF. Asbestos and colon cancer: a weight-of-the-evidence review. Environ Health Perspect. 1994;102:1038-50. |  |  |
| Asbestos exposure AND gastrointestinal cancer AND dose-response  (n=4) | | 1. | Goodman M, Morgan RW, Ray R, Malloy CD, Zhao K. Cancer in asbestos-exposed occupational cohorts: a meta-analysis. Cancer Causes Control. 1999;10:453-65. |  |  |
|  |  | 2. | Weiss W. The lack of causality between asbestos and colorectal cancer. J Occup Environ Med. 1995;37:1364-73. |  |  |
|  |  | 3. | Huncharek M. The biomedical and epidemiological characteristics of asbestos-related diseases: a review. Yale J Biol Med. 1986 ;59:435-51. |  |  |
|  |  | 4. | Levine DS. Does asbestos exposure cause gastrointestinal cancer? Dig Dis Sci. 1985;30:1189-98. |  |  |
