# Supplementary material for: Fiber burden and asbestos-related diseases: an umbrella review
Source: Gac Sanit. Author manuscript; Available in PMC 2022 Mar 27. (PMC8882348; doi:10.1016/j.gaceta.2021.04.001)
Supplement: Supplemental Table 5 [file NIHMS1776636-supplement-Supplemental_Table_5.doc]

**SYNTAXES AND WEBSITES**

**SYNTAXES USED IN THIS REVIEW**

First: “*asbestos exposure AND lung cancer*” OR *“asbestos exposure AND mesothelioma” OR “asbestos exposure AND asbestosis” OR “asbestos exposure AND diffuse interstitial pulmonary fibrosis” OR “asbestos exposure AND pleural plaques” OR “asbestos exposure AND diffuse pleural fibrosis” OR “asbestos exposure AND laryngeal cancer” OR “asbestos exposure AND gastrointestinal cancer”.*

Second: *“asbestos exposure AND lung cancer AND fiber concentration” OR “asbestos exposure AND mesothelioma AND fiber concentration” OR “asbestos exposure AND asbestosis AND fiber concentration” OR “asbestos exposure AND diffuse interstitial pulmonary fibrosis AND fiber concentration” OR “asbestos exposure AND pleural plaques AND fiber concentration” OR “asbestos exposure AND diffuse pleural fibrosis AND fiber concentration” OR “asbestos exposure AND laryngeal cancer AND fiber concentration” OR “asbestos exposure AND gastrointestinal cancer AND fiber concentration”.*

Third: *“asbestos exposure AND lung cancer AND dose-exposure” OR “asbestos exposure AND mesothelioma AND dose-exposure” OR “asbestos exposure AND asbestosis AND dose-exposure” OR “asbestos exposure AND diffuse interstitial pulmonary fibrosis AND dose-exposure” OR “asbestos exposure AND pleural plaques AND dose-exposure” OR “asbestos exposure AND diffuse pleural fibrosis AND dose-exposure” OR “asbestos exposure AND laryngeal cancer AND dose-exposure” OR “asbestos exposure AND gastrointestinal cancer AND dose-exposure”.*

Fourth: *“asbestos exposure AND lung cancer AND exposure-response” OR “asbestos exposure AND mesothelioma AND exposure-response” OR “asbestos exposure AND asbestosis AND exposure-response” OR “asbestos exposure AND diffuse interstitial pulmonary fibrosis AND exposure-response” OR “asbestos exposure AND pleural plaques AND exposure-response” OR “asbestos exposure AND diffuse pleural fibrosis AND exposure-response” OR “asbestos exposure AND laryngeal cancer AND exposure-response” OR “asbestos exposure AND gastrointestinal cancer AND exposure-response”.*

Fifth: *“asbestos exposure AND lung cancer AND dose-response” OR “asbestos exposure AND mesothelioma AND dose-response” OR “asbestos exposure AND asbestosis AND dose-response” OR “asbestos exposure AND diffuse interstitial pulmonary fibrosis AND dose-response” OR “asbestos exposure AND pleural plaques AND dose-response” OR “asbestos exposure AND diffuse pleural fibrosis AND dose-response” OR “asbestos exposure AND laryngeal cancer AND dose-response” OR “asbestos exposure AND gastrointestinal cancer AND dose-response”.*

Each of these syntaxes was applied independently and duplicate studies were eliminated. Titles and abstracts were screened using independent peer-review.

**WEBSITES CONSULTED IN THIS REVIEW**

1. International Agency for Research on Cancer (IARC) [Website]. Lyon (France): IARC monographs on the identification of carcinogenic hazards to humans. Volume 100C: Asbestos (chrysolite, amosite, crocidolite, tremolite, actinolite and anthophillite); 2012: pp219-309. [Cited 2021 March 01]. Available from: <https://monographs.iarc.fr/monographs-available/#25>
2. Ministerio de Trabajo, Migraciones y Seguridad Social. Instituto Nacional de Seguridad e Higiene en el Trabajo (INSHT) [Website]. Guía técnica para la evaluación y prevención de los riesgos relacionados con la exposición al amianto (RD 396/2006, BOE nº 86, de 11 de abril). [Cited 2021 April 01]. Available from: <https://cutt.ly/8ryw0z8>
3. Ministerio de Trabajo y Asuntos Sociales. Instituto Nacional de Seguridad e Higiene en el Trabajo (INSHT) [Website]. Nota Técnica de Prevención (NTP) 463: Exposición a fibras de amianto en ambientes interiores. 1995; [Cited 2021 April 01]. Available from: <https://cutt.ly/8ryw1G2>
4. American Conference of Governmental Industrial Hygienists (ACGIH) [Web site]. TLV/BEI Guidelines. [Updated on 2021]; [Cited 2021 January 8]. Available from: <https://www.acgih.org/tlv-bei-guidelines/policies-procedures-presentations/overview>
5. Occupational Safety and Health Administration (OSHA) [Website]. Fact Sheet: Asbestos. 2014; [Cited 2021 April 01]. Available from: https://www.osha.gov/Publications/OSHA3507.pdf
6. Official Journal of the European Union [Website]. Directive 2009/148/EC of the European Parliament and of the Council on the protection of workers from the risks related to exposure to asbestos at work. 2009; [Cited 2021 April 01]. Available from: https://cutt.ly/VrywMuC
7. Ministerio de Trabajo, Migraciones y Seguridad Social. Instituto Nacional de Seguridad, Salud en el Trabajo (INSST). Límites de exposición profesional para agentes químicos en España. 2019; [Cited 2021 April 01]. Available from: https://cutt.ly/BrywB2I
8. Agency for Toxic Substances and Disease Registry (ATSDR) [Website]. Asbestos. [Updated 2011 March 3]; [Cited 2021 April 01]. Available from: https://www.atsdr.cdc.gov/substances/toxsubstance.asp?toxid=4
9. United Estates Environmental Protection Agency (EPA) [Website]. Asbestos. [Updated 2019 April 16]; [Cited 2021 April 01]. Available from: <https://www.epa.gov/asbestos>
10. Health and Safety Executive (HSE) [Website]. Asbestos. [Cited 2021 April 01]. Available from: http://www.hse.gov.uk/asbestos/information.htm
11. Institut for Work and Health [Website]. Asbestos. [Updated 2019 December]; [Cited 2021 April 01]. Available from: <https://cutt.ly/LryrzTB>
12. Centers for Disease Control and prevention (CDC). The National Institute for Occupational Safety and Health (NIOSH). Appendix C - Supplementary Exposure Limits. [Cited 2021 January 8]. Available from: <https://www.cdc.gov/niosh/npg/nengapdxc.html>
13. Canadian Centre for Occupational Health and Safety (CCOHS). Federal government lowers limit of exposure to airborne chrysotile asbestos. [Cited 2021 January 8]. Available from: <https://www.ccohs.ca/oshanswers/chemicals/asbestos/control.html>
14. Health and Safety executive (HSE). Asbestos – FAQs: what is the control limit? [Cited 2021 January 8]. Available from: <https://www.hse.gov.uk/asbestos/faq.htm>
15. European Agency for Safety and Health at Work (EU-OSHA). Directive 2009/148/EC-exposure to asbestos at work. [Cited 2021 January 8]. Available from: <https://osha.europa.eu/en/legislation/directives/2009-148-ec-exposure-to-asbestos-at-work>
16. International Labour Organization (ILO) [Web site]. Thirteenth Session of the Joint ILO / WHO Committee on Occupational Health. Geneva, 9–12 December 2003. International Labor Office; 2006. Outline for the Development of National Programmes for Elimination of Asbestos-Related Diseases. Available in: <https://www.ilo.org/global/topics/safety-and-health-at-work/resources-library/publications/WCMS_108555/lang--en/index.htm>
