# Supplementary material for: Fiber burden and asbestos-related diseases: an umbrella review
Source: Gac Sanit. Author manuscript; Available in PMC 2022 Mar 27. (PMC8882348; doi:10.1016/j.gaceta.2021.04.001)
Supplement: Supplemental Table 2 [file NIHMS1776636-supplement-Supplemental_Table_2.doc]

| Supplementary Table 2. Number of reviews retrieved for each semantic search engine (syntax). | | |
| --- | --- | --- |
| **Search Strategies** | | **Nº Pre-Selected References (inclusion criteria)** |
| Asbestos exposure AND lung cancer (n=630) | Asbestos exposure AND lung cancer AND fiber concentration | 16 |
|  | Asbestos exposure AND lung cancer AND dose-exposure | 2 |
|  | Asbestos exposure AND lung cancer AND exposure-response | 13 |
|  | Asbestos exposure AND lung cancer AND dose-response | 24 |
| Asbestos exposure AND mesothelioma (n=767) | Asbestos exposure AND mesothelioma AND fiber concentration | 16 |
|  | Asbestos exposure AND mesothelioma AND dose-exposure | 4 |
|  | Asbestos exposure AND mesothelioma AND exposure-response | 11 |
|  | Asbestos exposure AND mesothelioma AND dose-response | 21 |
| Asbestos exposure AND asbestosis (n=323) | Asbestos exposure AND asbestosis AND fiber concentration | 11 |
|  | Asbestos exposure AND asbestosis AND dose-exposure | 1 |
|  | Asbestos exposure AND asbestosis AND exposure-response | 5 |
|  | Asbestos exposure AND asbestosis AND dose-response | 11 |
| Asbestos exposure AND diffuse interstitial pulmonary fibrosis (n=8) | Asbestos exposure AND diffuse interstitial pulmonary fibrosis AND fiber concentration | 5 |
|  | Asbestos exposure AND diffuse interstitial pulmonary fibrosis AND dose-exposure | 0 |
|  | Asbestos exposure AND diffuse interstitial pulmonary fibrosis AND exposure-response | 0 |
|  | Asbestos exposure AND diffuse interstitial pulmonary fibrosis AND dose-response | 2 |
| Asbestos exposure AND pleural plaques (n=86) | Asbestos exposure AND pleural plaques AND fiber concentration | 10 |
|  | Asbestos exposure AND pleural plaques AND dose-exposure | 0 |
|  | Asbestos exposure AND pleural plaques AND exposure-response | 2 |
|  | Asbestos exposure AND pleural plaques AND dose-response | 4 |
| Asbestos exposure AND diffuse pleural fibrosis  (n=22) | Asbestos exposure AND diffuse pleural fibrosis AND fiber concentration | 1 |
|  | Asbestos exposure AND diffuse pleural fibrosis AND dose-exposure | 0 |
|  | Asbestos exposure AND diffuse pleural fibrosis AND exposure-response | 0 |
|  | Asbestos exposure AND diffuse pleural fibrosis AND dose-response | 0 |
| Asbestos exposure AND laryngeal cancer (n=18) | Asbestos exposure AND laryngeal cancer AND fiber concentration | 0 |
|  | Asbestos exposure AND laryngeal cancer AND dose-exposure | 0 |
|  | Asbestos exposure AND laryngeal cancer AND exposure-response | 0 |
|  | Asbestos exposure AND laryngeal cancer AND dose-response | 2 |
| Asbestos exposure AND gastrointestinal cancer  (n=38) | Asbestos exposure AND gastrointestinal cancer AND fiber concentration | 3 |
|  | Asbestos exposure AND gastrointestinal cancer AND dose-exposure | 0 |
|  | Asbestos exposure AND gastrointestinal cancer AND exposure-response | 2 |
|  | Asbestos exposure AND gastrointestinal cancer AND dose-response | 4 |
| **Total articles retrieved** | | **170** |
